# Redistribution of H3K27me3 upon DNA hypomethylation results in de-repression of Polycomb target genes

**DOI:** 10.1186/gb-2013-14-3-r25

**Published:** 2013-03-25

**Authors:** James P Reddington, Sara M Perricone, Colm E Nestor, Judith Reichmann, Neil A Youngson, Masako Suzuki, Diana Reinhardt, Donncha S Dunican, James G Prendergast, Heidi Mjoseng, Bernard H Ramsahoye, Emma Whitelaw, John M Greally, Ian R Adams, Wendy A Bickmore, Richard R Meehan

**Affiliations:** 1 MRC Human Genetics Unit, MRC Institute of Genetics and Molecular Medicine, University of Edinburgh, Edinburgh EH4 2XU, UK; 2 Breakthrough Breast Cancer Research Unit, University of Edinburgh, Western General Hospital, Edinburgh EH4 2XU, UK; 3 Queensland Institute of Medical Research, Herston, Queensland 4006, Australia; 4 Departments of Genetics (Computational Genetics) and Center for Epigenomics, Albert Einstein College of Medicine, 1301 Morris Park Avenue, Bronx, NY, USA; 5 Institute of Genetics and Molecular Medicine, University of Edinburgh, Edinburgh EH4 2XU, UK

**Keywords:** DNA methylation, H3K27me3, Polycomb, PRC2, regulation of transcription

## Abstract

**Background:**

DNA methylation and the Polycomb repression system are epigenetic mechanisms that play important roles in maintaining transcriptional repression. Recent evidence suggests that DNA methylation can attenuate the binding of Polycomb protein components to chromatin and thus plays a role in determining their genomic targeting. However, whether this role of DNA methylation is important in the context of transcriptional regulation is unclear.

**Results:**

By genome-wide mapping of the Polycomb Repressive Complex 2-signature histone mark, H3K27me3, in severely DNA hypomethylated mouse somatic cells, we show that hypomethylation leads to widespread H3K27me3 redistribution, in a manner that reflects the local DNA methylation status in wild-type cells. Unexpectedly, we observe striking loss of H3K27me3 and Polycomb Repressive Complex 2 from Polycomb target gene promoters in DNA hypomethylated cells, including *Hox *gene clusters. Importantly, we show that many of these genes become ectopically expressed in DNA hypomethylated cells, consistent with loss of Polycomb-mediated repression.

**Conclusions:**

An intact DNA methylome is required for appropriate Polycomb-mediated gene repression by constraining Polycomb Repressive Complex 2 targeting. These observations identify a previously unappreciated role for DNA methylation in gene regulation and therefore influence our understanding of how this epigenetic mechanism contributes to normal development and disease.

## Background

Epigenetic mechanisms, such as DNA methylation and the Polycomb repressor system, play key roles in maintaining transcriptional states that are initially established by transcription factor networks [1,2]. A major challenge of molecular biology is to understand how epigenetic mechanisms contribute to the precise temporal and spatial patterns of gene expression that are required for multicellular life, and how the malfunction of these mechanisms contributes to human disease.

DNA methylation involves the addition of a methyl group to position 5 of the pyrimidine ring of the cytosine base, a reaction catalyzed by a family of DNA methyltransferase enzymes [1]. In mammals, DNA methylation occurs predominantly in the sequence context of 5′-CG-3′ (CpG) [3,4]. Vertebrates possess a so-called global methylome, as in most tissues, the majority of cytosines in the CpG context are found in the methylated state (5mCpG) [3,5]. The high level of CpG methylation found in the bulk genome is punctuated by short stretches of CpG- and GC-rich sequences, known as CpG islands, that are normally infrequently methylated and are associated with a large proportion of gene promoters [1,3,6]. Despite this general bimodality, DNA methylation patterns are variable between cells of different tissues, and dynamic during cell differentiation, a feature thought to contribute to the maintenance of a cell's transcriptional state [3-5,7]. At gene promoters that contain sufficient CpG density, abundant 5mCpG is associated with transcriptional repression [8]. This canonical regulatory role for DNA methylation contributes to the monoallelic repression of imprinted genes [9], the stable repression of large regions of the inactive X-chromosome in female cells [10], and the tissue-specific repression of a relatively small number of single copy genes [11,12]. For example, promoter DNA methylation is utilized in somatic cells to maintain repression of genes that are expressed in the germline [13-15]. DNA methylation is able to contribute to the maintenance of transcription states over time and cell divisions through two main properties. Firstly, patterns of 5mCpG are copied to the nascent strand during replication of DNA in a process requiring the maintenance DNA methyltransferase, Dnmt1 [1]. This property means that patterns of 5mCpG are stable over cell division. Secondly, CpG and 5mCpG are differentially bound by various DNA- and/or chromatin-binding proteins, many of which have key roles in transcriptional regulation [1,16]. Several of these proteins are either directly or indirectly involved in further chromatin modification, leading to the idea that DNA methylation acts as a template to direct the establishment or reinforcement of chromatin states. For example, 5mCpG is specifically recognized by methyl-CpG-binding proteins, a number of which are associated with histone deacetylases and other histone-modifying enzymes [1,17,18]. By contrast, unmodified CpG is recognized by proteins such as the histone lysine demethylase Kdm2a, and Cfp1, a protein that recruits a Setd1 H3K4 methyltransferase complex [19,20]. Despite advances in this area, our understanding of how DNA methylation contributes to chromatin states, and how these states influence gene regulation, is far from complete.

Polycomb group proteins form multi-protein chromatin-associated complexes that act as repressors of thousands of genes, many of which have key functions in embryonic development and cell-fate decisions [21-24]. The Polycomb Repressor Complex 2 (PRC2) modifies chromatin structure by depositing tri-methylation of lysine 27 on histone H3 (H3K27me3) via its catalytic Ezh2/Ezh1 subunit [2,25]. The H3K27me3 mark is therefore considered a hallmark of PRC2-mediated repression [2]. Like DNA methylation, H3K27me3 is propagated through cell divisions, a process mediated by the ability of the PRC2 complex to bind to H3K27me3 [26,27]. Evidence suggests that H3K27me3 and PRC2 lead to transcriptional silencing in multiple ways, including the recruitment of a subset of Polycomb Repressor Complex 1 (PRC1) protein complexes that recognize the H3K27me3 mark and induce chromatin compaction [2,28,29].

In the last few years it has emerged that considerable cross-talk exists between the DNA methylation and Polycomb repression systems, demonstrating that these two epigenetic mechanisms are intimately linked. Multiple lines of evidence suggest that DNA methylation is a negative modulator of PRC2-chromatin interactions. Firstly, methylation-free CpG islands have been linked to the recruitment of the PRC2 complex [30-32]. Secondly, epigenome mapping studies have found a negative correlation between DNA methylation and H3K27me3 patterns in normal [3,5,7,33] and cancer [34,35] tissues, implying exclusivity of the two marks in mammalian genomes. A similar observation has been made in plant tissues, implying deep conservation of this relationship [36]. It should be noted that, in mammals, a negative correlation between DNA methylation and H3K27me3 is restricted to relatively CpG-rich regions of the genome, whereas the two marks co-occupy many CpG-poor regions [37,38]. The idea of exclusivity is supported by *in vitro *experiments demonstrating that PRC2 shows attenuated binding and/or activity on DNA methylated chromatin, suggesting that 5mC directly influences PRC2-chromatin interactions [39,40]. Importantly, when DNA methylation patterns are experimentally perturbed, H3K27me3 patterns are altered [32,33,37,39]. For example, in mouse embryonic stem cells that are hypomethylated due to lack of DNA methyltransferases, new domains of H3K27me3 form at genomic regions that are normally highly DNA methylated in wild-type cells [22,32]. Also, in hypomethylated mouse embryonic stem cells, H3K27me3 is reduced at regions where it is normally abundant in wild-type cells [37].

Collectively, these studies have demonstrated that DNA methylation plays a role in ensuring correct genomic targeting of the PRC2 complex and hence the distribution of the H3K27me3 mark on chromatin. However, it is unknown if this function of DNA methylation is important in the context of transcriptional regulation. We reasoned that perturbations in DNA methylation may lead to altered patterns of transcription due to redistribution of the repressive activity of the PRC2 complex and the H3K27me3 mark. To test this hypothesis, we generated genome-wide profiles of H3K27me3, gene expression and DNA methylation in severely DNA hypomethylated mouse somatic cells. Our results uncover an unexpected relationship between DNA methylation and transcriptional repression by Polycomb. We suggest that, through facilitating correct PRC2-targeting, an intact DNA methylome is required for appropriate Polycomb-mediated gene repression.

## Results

### Mapping epigenetic marks in severely DNA hypomethylated *Dnmt1^-/- ^*mouse somatic cells

**Figure 1:**
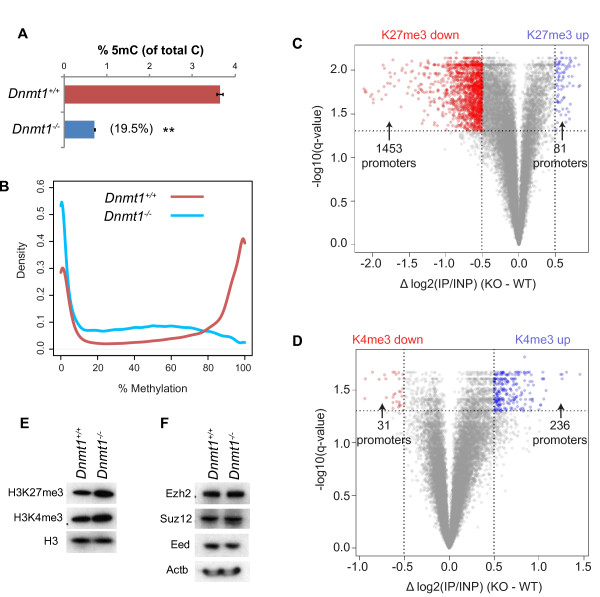
**H3K27me3 and H3K4me3 mapping at gene promoters in DNA hypomethylated somatic cells**. **(A) **Global quantification of the 5 mC base by HPLC. Values are 5 mC as a percentage of total C in *Dnmt1^+/+ ^*and *Dnmt1^-/- ^*MEFs. Error bars represent standard error of the mean. **P <0.001 by t-test. **(B) **Density plots showing CpG methylation levels in *Dnmt1^+/+ ^*and *Dnmt1^-/- ^*MEFs as measured by RRBS. **(C, D) **Volcano plots of all promoter H3K27me3 (C) and H3K4me3 (D) ChIP data measured by promoter microarray (ChIP-chip). Difference in normalized average promoter values between *Dnmt1^+/+ ^*and *Dnmt1^-/- ^*MEFs (Δlog2(IP/INP)) is plotted against q-value (*P*-value (two-tailed t-test) corrected for multiple testing by Benjamini-Hochberg method). Promoters that have a difference of <-0.5 or >0.5 and a q-value <0.05 are defined as significantly different. Red points indicate promoters with reduced H3K27me3/H3K4me3 in *Dnmt1^-/- ^*MEFs (H3K27me3/H3K4me3 down) and blue indicate promoters with increased H3K27me3/H3K4me3 in *Dnmt1^-/- ^*MEFs (H3K27me3/H3K4me3 up). **(E, F) **Western blot for histone modifications (E) and core PRC2 components (F) in *Dnmt1^+/+ ^*and *Dnmt1^-/- ^*MEFs. mC, methyl-cytosine. KO, knock-out, *Dnmt1^-/- ^*MEFs. WT, wild-type, *Dnmt1^+/+ ^*MEFs.

As a first step to investigate the interplay between DNA methylation and PRC2 in the context of gene regulation, we generated maps of DNA methylation, H3K4me3 and the PRC2-signature H3K27me3 histone mark in cells where DNA methylation is strongly reduced. We utilized a previously developed genetic system to induce DNA hypomethylation, using mouse embryonic fibroblasts (MEFs) that are homozygous for a hypomorphic allele of *Dnmt1 *(*Dnmt1^n/n^*), the gene encoding the major maintenance DNA methyltransferase [12,41]. In addition to a *Dnmt1 *mutation, these cells are homozygous null for *trp53*, encoding the p53 protein, and are therefore compared to *trp53^-/- ^*cells as control (herein referred to as *Dnmt1^+/+ ^*and *Dnmt1^-/- ^*MEFs for simplicity) [12]. The advantage of this system is that *Dnmt1^-/- ^*MEFs are severely hypomethylated yet viable in culture [12]. We quantified global levels of the 5mC base by performing high performance liquid chromatography (HPLC) (Figure 1A). This assay confirmed that *Dnmt1^-/- ^*MEFs are severely hypomethylated at the global level, possessing <20% residual 5mC compared to controls (Figure 1A; *P *<0.001 by t-test). We generated single-nucleotide resolution maps of DNA methylation from *Dnmt1^+/+ ^*and *Dnmt1^-/- ^*MEFs by extended-coverage reduced-representation bisulfite sequencing (RRBS) (see Materials and methods). Using this method we quantified the methylation level of >1.7 million CpGs that had a sequencing depth of at least 10 reads in each condition (the median depth was >26 reads among these CpGs in each condition). In *Dnmt1^+/+ ^*MEFs, the expected bimodal distribution of CpG methylation was observed, with most CpGs having either very high or very low levels of DNA methylation (Figure 1B). In *Dnmt1^-/- ^*MEFs, CpG methylation was strongly reduced consistent with widespread hypomethylation (Figure 1B).

We next examined the effect of DNA hypomethylation on H3K27me3 and H3K4me3 patterns at gene promoter regions. We initially used native chromatin-immunoprecipitation (ChIP) [28,42] coupled to a microarray that represents over 24,000 RefSeq gene promoters (ChIP-chip approach). We validated the accuracy of this approach using real-time quantitative PCR (ChIP-qPCR) for nine selected promoter regions (Figure S1 in Additional file 1). Enrichments using these two methods were highly similar (H3K27me3, R^2 ^= 0.826, *P *<0.001; H3K4me3, R^2 ^= 0.876, *P *<0.001). Using the ChIP-chip data, we identified promoters that were differentially marked by either histone modification in hypomethylated cells by selecting those where the q-value (t-test *P*-value corrected for multiple testing by the Benjamini-Hochberg method [43]) was less than 0.05 and the difference between the mean promoter enrichment (expressed as the log2 ratio of immunoprecipitate (IP) signal to input (INP) signal - log2(IP/INP)) across conditions was greater than 0.5 in either direction (Figure 1C,D). We observed a large number of promoters that showed differential H3K27me3 or H3K4me3 in hypomethylated cells, consistent with the notion that DNA methylation is an important modulator of these histone marks. The data for all analyzed promoter regions is supplied as a supplementary file (Additional file 2). Surprisingly, we observed 1,453 promoter regions with significantly lower H3K27me3 in *Dnmt1^-/- ^*compared to *Dnmt1^+/+ ^*MEFs ('H3K27me3 down' promoters) (Figure 1C). We observed that 81 promoters showed the opposite trend, with increased H3K27me3 in *Dnmt1^-/- ^*MEFs ('H3K27me3 up' promoters) (Figure 1C). For H3K4me3, 236 promoters were found to have increased enrichment in *Dnmt1^-/- ^*MEFs ('H3K4me3 up' promoters) whereas 31 showed lower enrichment ('H3K4me3 down' promoters) (Figure 1D). Importantly, immunoblotting showed that there was no global decrease in the H3K27me3 mark or the protein levels of the core PRC2 subunits in hypomethylated cells, suggesting that the observed changes in H3K27me3 distribution were not due to loss of PRC2 function in these cells (Figure 1E,F). In fact, the global levels of H3K27me3 and H3K4me3 appeared to be increased in *Dnmt1^-/- ^*relative to *Dnmt1^+/+ ^*MEFs, consistent with DNA methylation having an inhibitory effect on the deposition of these histone marks [19,39].

Because we observed a large number of promoters that lose H3K27me3 in *Dnmt1^-/- ^*MEFs (Figure 1C), only a small number of promoters that gain this mark (Figure 1C), and no decrease in global H3K27me3 (Figure 1E), we hypothesized that H3K27me3 increases at regions outside of gene promoters in hypomethylated cells. Indeed, it has recently been observed that, in embryonic stem (ES) cells deficient for DNA methyltransferases, increased H3K27me3 occurs over large domains [32,37]. To address this possibility, we mapped the H3K27me3 and H3K4me3 histone marks genome-wide using ChIP followed by massively parallel sequencing (ChIP-seq) from *Dnmt1^+/+ ^*and *Dnmt1^-/- ^*MEFs. This assay replicated the changes observed at promoters by ChIP-chip, and in addition identified regions that showed differential enrichment for H3K27me3 between *Dnmt1^-/- ^*and *Dnmt1^+/+ ^*MEFs in a genome-wide manner (see Materials and methods). We observed increased H3K27me3 in hypomethylated cells at many regions outside of gene promoters, consistent with our prediction. Genomic regions found to differ in H3K27me3 between the two cell lines are provided as a supplementary file (100 kb genomic windows: Additional file 3; 1 kb genomic windows: Additional file 4). Two intergenic regions are shown in Figure S2 in Additional file 1, where increased H3K27me3 was observed over large domains in *Dnmt1^-/- ^*MEFs compared to low enrichments in *Dnmt1^+/+ ^*MEFs. We used ChIP-qPCR to confirm the observed increased H3K27me3 at these regions in *Dnmt1^-/- ^*MEFs (Figure S2C in Additional file 1).

**Figure 2:**
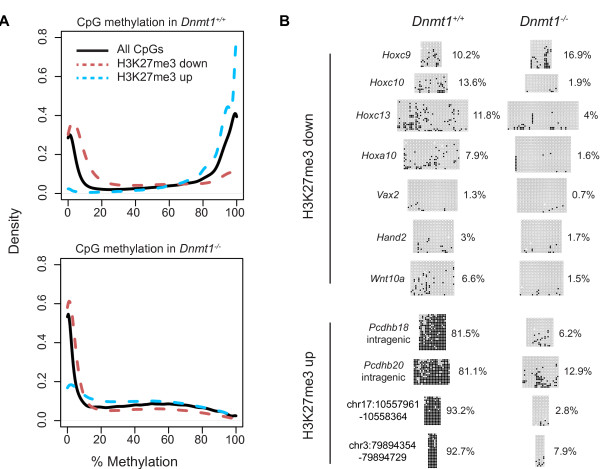
**H3K27me3 redistribution in hypomethylated cells is associated with DNA methylation pattern. (A) **Density plots showing the methylation of CpGs in *Dnmt1^+/+ ^*(upper) and *Dnmt1^-/- ^*(lower) MEFs. The methylation is plotted for all CpGs, CpGs within H3K27me3 down regions and H3K27me3 up regions defined by ChIP-seq. **(B) **Bisulfite sequencing of selected H3K27me3 down and H3K27me3 up regions in *Dnmt1^+/+ ^*and *Dnmt1^-/- ^*MEFs. Filled circles (black) represent 5mCpG and open circles represent CpG. Gaps indicate inconclusive base calling at a CpG site. The overall amount of 5mCpG as a percentage of total CpG in each amplicon is shown adjacent to each image. H3K27me3 down regions represent proximal promoter regions while H3K27me3 up regions represent intragenic or intergenic regions.

As DNA methylation is thought to have a negative effect on the binding or activity of the PRC2 complex on chromatin [39,40], we reasoned that if the observed changes in H3K27me3 occupancy in DNA hypomethylated somatic cells are directly caused by changes in DNA methylation, then they should reflect local DNA methylation levels. For example, H3K27me3 up regions would be expected to be highly DNA methylated in *Dnmt1^+/+ ^*cells, where DNA methylation inhibits H3K27me3 deposition, and hypomethylated in *Dnmt1^+/+ ^*cells, where H3K27me3 is increased. Conversely, H3K27me3 down regions may be associated with low levels of DNA methylation in *Dnmt1^+/+ ^*cells where they are enriched for H3K27me3. We tested this hypothesis by examining DNA methylation levels in RRBS data of CpGs within regions that showed increased or decreased H3K27me3 in DNA hypomethylated cells by ChIP-seq. In *Dnmt1^+/+ ^*MEFs, all CpGs showed the expected bimodal distribution of DNA methylation levels (Figure 2A upper). CpGs within H3K27me3 down regions had low levels of methylation, whereas CpGs within H3K27me3 up regions had high levels of DNA methylation (Figure 2A upper). In *Dnmt1^-/- ^*cells, the DNA methylation level of CpGs in all of these categories was decreased (Figure 2A lower). The same associations between H3K27me3 changes and DNA methylation levels in *Dnmt1^+/+ ^*MEFs were observed when DNA methylation was mapped using an independent method, *HpaII *tiny fragment enrichment by ligation-mediated PCR (HELP) followed by tag sequencing (HELP-tag-seq) (Figure S3 in Additional file 1). We performed bisulfite sequencing of selected H3K27me3 down and up regions to confirm that they were associated with low and high levels of DNA methylation, respectively, in wild-type cells (Figure 2B).

These observations support the notion that DNA methylation is an important modulator of H3K27me3 patterns, and demonstrate that severe DNA hypomethylation leads to altered distribution of H3K27me3 in mouse somatic cells, suggesting that this is not an exclusive feature of hypomethylated mouse ES cells. Importantly, this map of H3K27me3 changes in DNA hypomethylated cells could be used as a starting point to investigate the interplay between DNA methylation and PRC2 in the context of transcriptional regulation.

### An intact DNA methylome is required for efficient binding of PRC2 to its normal target gene promoters including *Homeobox *gene clusters

**Figure 3:**
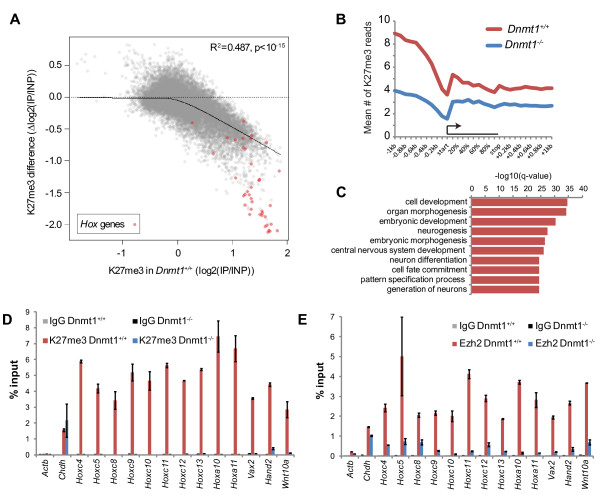
**H3K27me3 is lost from Polycomb target gene promoters in DNA hypomethylated somatic cells**. **(A) **Scatter plot showing the relationship between H3K27me3 enrichment in *Dnmt1^+/+ ^*MEFs (x-axis) and the difference in H3K27me3 enrichments between *Dnmt1^+/+ ^*and *Dnmt1^-/- ^*MEFs (y-axis) at all promoter regions by ChIP-chip. A llocally weighted scatterplot smoothing line (black) is plotted. The R^2 ^value (Pearson's) is shown alongside the *P*-value from a Pearson's correlation test. *Hox *gene promoters are plotted as red points. **(B) **H3K27me3 ChIP-seq data for regions surrounding Polycomb target genes. Polycomb target genes were defined as the top 10% of gene promoters as ranked by H3K27me3 enrichment (by ChIP-chip) in *Dnmt1^+/+ ^*MEFs. **(C) **Top 10 Gene Ontology terms (Biological Process) found to be significantly enriched among H3K27me3 down genes using all genes covered on the microarray as background. The q-value for enrichment of each term is plotted as -log10(q-value). **(D, E) **ChIP-qPCR for H3K27me3 (D) and Ezh2 (E) in *Dnmt1^+/+ ^*and *Dnmt1^-/- ^*MEFs using primers for the promoters of the indicated genes. Enrichment for each immunoprecipitation (IP) is expressed as percentage of DNA in input. Non-specific IgG IP and the *Actb *promoter are shown as negative controls and the *Chdh *promoter is shown as a positive control. Error bars indicate ± standard error of the mean of two experiments.

The most surprising facet of H3K27me3 redistribution in hypomethylated somatic cells was that this mark was lost from a large number of gene promoters (Figure 1C). We reasoned that, due to increased binding of the PRC2 complex to numerous newly uncovered sites within a hypomethylated genome, the complex may be diluted from its normal targets. We looked for a relationship between promoter H3K27me3 enrichment in *Dnmt1^+/+ ^*MEFs and H3K27me3 difference between *Dnmt1^+/+ ^*and *Dnmt1^-/- ^*MEFs, and found that they were negatively correlated (Figure 3A; R^2 ^= 0.487, *P *<10^-15^). Thus, promoters that represented normal Polycomb targets in *Dnmt1^+/+ ^*MEFs lost H3K27me3 in *Dnmt1^-/- ^*MEFs. For example, most promoters from the four *Hox *gene clusters, classic Polycomb target genes, exhibited striking loss of H3K27me3 upon hypomethylation (Figure 3A, red points). We performed a pile-up of sequence reads from ChIP-seq at genes that were highly marked by H3K27me3 in *Dnmt1^+/+ ^*MEFs (the top 10% of genes measured by ChIP-chip, Figure 3B). As expected, these genes showed enrichment for H3K27me3 reads in *Dnmt1^+/+ ^*MEFs, particularly in their upstream regions. In *Dnmt1^-/- ^*MEFs, the H3K27me3 profile was lower across the whole region analyzed, consistent with loss of PRC2 function at its normal target genes in *Dnmt1^-/- ^*MEFs (Figure 3B). We tested for enrichment in Gene Ontology terms among H3K27me3 down genes and found that they were highly enriched for functions in cell development, embryonic development and cell-fate commitment, reflecting terms that have previously been associated with Polycomb target genes (Figure 3C) [22-24,44]. Indeed, when H3K27me3 target genes were used as a background for Gene Ontology enrichment analysis of H3K27me3 down genes, no significant terms were found. Together, these results suggest that, in hypomethylated cells, H3K27me3 is not lost from a specific set of Polycomb target genes but rather it is generally decreased at its normal target promoters, consistent with a 'dilution' hypothesis.

We performed ChIP-qPCR using primers designed for the promoter regions of selected H3K27me3 down genes, including *Hox *genes, to validate the H3K27me3 loss in *Dnmt1^-/- ^*MEFs that was observed by ChIP-chip. The *Actb *(negative) and *Chdh *(positive) promoters were included as controls for the assay. All of the selected H3K27me3 down promoters showed striking loss of H3K27me3 enrichment in *Dnmt1^-/- ^*MEFs, down to levels comparable to the negative control *Actb *promoter and non-specific immunoglobulin G (IgG) immunoprecipitation (Figure 3D). Of note, several adjacent *HoxC *genes were found to have lost H3K27me3 enrichment in *Dnmt1^-/- ^*MEFs (Figure 3D). To determine whether H3K27me3 loss in *Dnmt1^-/- ^*MEFs was concomitant with loss of PRC2 binding to these gene promoters, we performed cross-linked ChIP for the PRC2 histone methyltransferase Ezh2 (Figure 3E). A marked reduction of Ezh2 binding at these gene promoters was observed in *Dnmt1^-/- ^*MEFs, consistent with loss of PRC2 binding (Figure 3E).

### De-repression of Polycomb target genes upon genome-wide loss of DNA methylation

**Figure 4:**
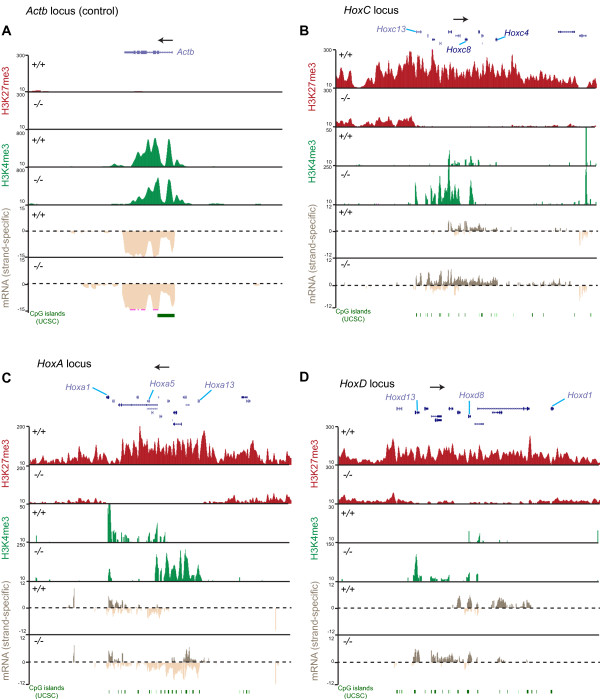
***Hox *gene clusters are mis-regulated in *Dnmt1^-/- ^*mouse embryonic fibroblasts**. Browser shots from the UCSC genome browser showing H3K27me3 (red) and H3K4me3 (green) ChIP-seq data, and strand-specific mRNA-seq data for the **(A) ***Actb*, **(B) ***HoxC*, **(C) ***HoxA *and **(D) ***HoxD *loci in *Dnmt1^+/+ ^*and *Dnmt1^-/- ^*MEFs. The position of RefSeq genes (blue) is shown along with the direction of sense transcription for the *Hox *genes (black arrow). The position of UCSC CpG islands (green) is shown. ChIP-seq data is represented as the number of reads that overlap genomic windows. Note the different scales used for H3K4me3 data. mRNA-seq data is represented as the number of reads that overlap genomic windows (log2) on each strand (above and below the dashed line).

As PRC2 and its associated histone methyltransferase activity are known to have key roles in transcriptional repression, we next asked if loss of H3K27me3 and PRC2 binding in *Dnmt1^-/- ^*MEFs is associated with ectopic gene expression. We generated transcriptome profiles of *Dnmt1^+/+ ^*and *Dnmt1^-/- ^*MEFs using strand-specific mRNA-seq and gene expression microarrays. Using these methods, *Dnmt1^-/- ^*MEFs showed the expected de-repression of genes known to rely on DNA methylation for repression, such as imprinted genes (data not shown). The *Actb *locus is shown in Figure 4A as a control as it was expected to be positive for H3K4me3 and expressed in both conditions and negative for H3K27me3. We first examined *Hox *gene clusters as they represent classic Polycomb target genes, are known to require PRC2 and/or H3K27me3 for their normal regulation [22,24], and were identified in our ChIP-chip as showing loss of H3K27me3 in *Dnmt1^-/- ^*MEFs. In *Dnmt1^+/+ ^*MEFs, the *HoxC *cluster was covered by a large block of H3K27me3, as previously reported for MEFs [45], whereas, strikingly, this block was absent in *Dnmt1^-/- ^*MEFs (Figure 4B). In *Dnmt1^+/+ ^*MEFs, RNA was detected on the sense strand in the region from *Hoxc4 *to *Hoxc10 *genes, whereas the *Hoxc11 *to *Hoxc13 *genes were silent (Figure 4B). In *Dnmt1^-/- ^*MEFs, concomitant with the loss of H3K27me3, RNA was detected on the sense strand from all genes in the cluster. Similar transcriptional effects at *Hox *gene clusters have been observed previously when Polycomb components are mutated in MEFs, consistent with the hypothesis that *Hox *gene mis-regulation in *Dnmt1^-/- ^*MEFs is caused by loss of Polycomb-mediated repression [46]. In addition to the de-repression of the *Hoxc11 *to *Hoxc13 *genes in *Dnmt1^-/- ^*MEFs, RNA was detected from intergenic regions and from the antisense strand. H3K4me3 was increased across much of the *HoxC *cluster in *Dnmt1^-/- ^*MEFs, particularly across the region of the cluster that becomes strongly de-repressed (Figure 4B). A similar effect was observed at the *HoxA *and *HoxD *clusters, including loss of H3K27me3 from a large genomic region and de-repression of *Hox *genes within the normally silent portion of the cluster (Figure 4C,D). The *HoxB *cluster showed loss of H3K27me3 across a large domain but did not show upregulation of RNA or H3K4me3 in *Dnmt1^-/- ^*MEFs (data not shown). Importantly, we have already shown that the promoters of the studied *Hox *genes, and H3K27me3 down regions in general, are associated with low levels of DNA methylation in *Dnmt1^+/+ ^*MEFs, suggesting that their de-repression in *Dnmt1^-/- ^*MEFs is not due to loss of canonical promoter DNA methylation-mediated repression (Figure 2). Instead, these observations are consistent with loss of PRC2-mediated repression at *Hox *genes in *Dnmt1^-/- ^*MEFs.

**Figure 5:**
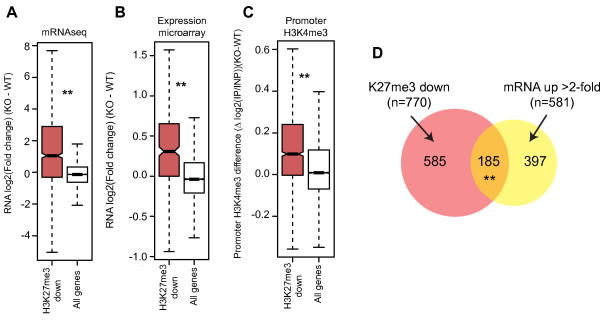
**H3K27me3 loss in hypomethylated cells is associated with ectopic gene expression**. **(A, B) **Boxplots displaying the relative expression (log2) of H3K27me3 down genes (red) and all genes (white) between *Dnmt1^+/+ ^*and *Dnmt1^-/- ^*MEFs as measured by mRNA-seq (A) and expression microarray (B). **P <0.001 by Wilcoxon rank sum test. **(C) **Boxplot showing the difference in promoter enrichment for H3K4me3 between *Dnmt1^+/+ ^*and *Dnmt1^-/- ^*MEFs as measured by ChIP-chip. **P <0.001 by Wilcoxon rank sum test. **(D) **Venn diagram showing overlap between H3K27me3 down genes (red circle) and genes that show >2-fold increased expression in *Dnmt1^-/- ^*relative to *Dnmt1^+/+ ^*MEFs by mRNA-seq (yellow circle). The number of genes in each section of the diagram is shown. **P <0.001 as estimated by hyper-geometric testing for the enrichment of the overlap genes.

As H3K27me3 is lost from a large number of gene promoters in *Dnmt1^-/- ^*MEFs, we investigated differential gene expression in these cells in a global manner using mRNA-seq and expression microarray. Using both methods, we observed that H3K27me3 down genes are associated with increased expression in *Dnmt1^-/- ^*MEFs (Figure 5A,B; *P *<0.001 by Wilcoxon rank sum test). We performed RT-qPCR to confirm upregulation at the mRNA level of selected H3K27me3 down genes in *Dnmt1^-/- ^*MEFs, including *Hox *genes (Figure S4 in Additional file 1). Using our promoter ChIP-chip data, we observed that H3K27me3 down genes were also associated with increased promoter H3K4me3 in *Dnmt1^-/- ^*MEFs, consistent with increased transcriptional initiation (Figure 5C; *P *<0.001 by Wilcoxon rank sum test). By overlapping H3K27me3 down genes and >2-fold upregulated genes (by mRNA-seq), we observed that 185 genes fell into both categories, a far greater overlap than would be expected by chance (Figure 5D; *P *<0.001 estimated by hyper-geometric testing). This represents around a third of the increased expression observed in hypomethylated cells, suggesting that contributing to Polycomb-mediated repression is a major function for DNA methylation in gene regulation. This also shows that only a proportion of genes that lose promoter H3K27me3 in DNA hypomethylated cells are transcriptionally upregulated, highlighting the involvement of other factors (for example, the presence or absence of certain transcription factors) in their regulation.

We wondered whether the increased H3K27me3 that is observed at certain genomic regions in DNA hypomethylated cells is associated with *de novo *repression of transcription. DNA methylation is high within many gene body regions and has been suggested to correlate positively with transcription and therefore may act to inhibit PRC2 binding [3,4,39]. We identified large genomic windows (100 kb) that showed increased or decreased H3K27me3 in *Dnmt1^-/- ^*MEFs by ChIP-seq (see Materials and methods) and examined the expression of genes within these windows using mRNA-seq. Genes within large H3K27me3 down windows were associated with increased expression in *Dnmt1^-/- ^*relative to *Dnmt1^+/+ ^*MEFs, consistent with our observations at gene promoter regions (Figure S5A in Additional file 1). Conversely, genes within large H3K27me3 up windows were associated with decreased expression in *Dnmt1^-/- ^*MEFs, consistent with increased PRC2-mediated repression at these genes in DNA hypomethylated cells (Figure S5A in Additional file 1). We chose two genes, *Pcdhb18 *and *Pcdhb20*, that we had already shown by bisulfite sequencing to be associated with dense DNA methylation within an intragenic CpG island in *Dnmt1^+/+ ^*MEFs (Figure 2B). We performed H3K27me3 ChIP-qPCR for each intragenic region, which showed that they were associated with increased H3K27me3 in *Dnmt1^-/- ^*MEFs when the DNA methylation is removed (Figure S5B in Additional file 1). We showed by qRT-PCR that the increased H3K27me3 at these genes was associated with decreased mRNA expression in *Dnmt1^-/- ^*MEFs (Figure S5C in Additional file 1).

These findings suggest that altered H3K27me3 distribution upon loss of DNA methylation is associated with changes in gene expression. Given that DNA methylation can directly modulate H3K27me3 patterns [39], and H3K27me3 and PRC2 are known to induce transcriptional repression, it is likely that redistribution of the repressive activity of PRC2 accounts for many of the observed transcriptional changes in DNA hypomethylated cells. However, it is not possible at this point to say that redistribution of H3K27me3 is the sole driver of the observed expression changes, as other factors are likely to contribute to gene expression changes in *Dnmt1^-/- ^*MEFs.

### Independent methods of inducing DNA methylation loss result in H3K237me3 redistribution and de-repression of Polycomb target genes

**Figure 6:**
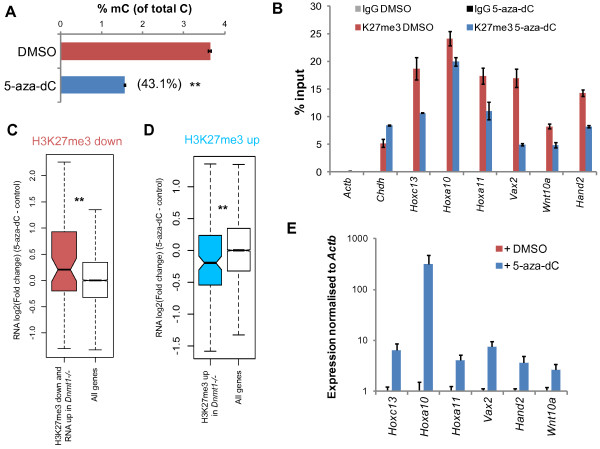
**Demethylation with a DNA methyltransferase inhibitor results in redistribution of H3K27me3**. **(A) **Global quantification of the 5mC base by HPLC. Values are 5 mC as a percentage of total C in *Dnmt1^+/+ ^*MEFs treated with vector control or 5-aza-dC. Error bars represent standard error of the mean (SEM). **P <0.001 by t-test. **(B) **ChIP-qPCR for H3K27me3 in *Dnmt1^+/+ ^*MEFs treated with vector control or 5-aza-dC using primers for the promoters of the indicated genes. Enrichment for each immunoprecipitation (IP) is expressed as percentage of DNA in input. Non-specific IgG IP and the *Actb *promoter are shown as negative controls and the *Chdh *promoter is shown as a positive control. Error bars indicate ±SEM of two experiments. **(C, D) **Boxplots showing the relative expression (log2) of the indicated gene set, and all genes, between vector control and treatment with 5-aza-dC in *Dnmt1^+/+ ^*MEFs. The gene sets compared are those that are both H3K27me3 down and >1.5 fold upregulated at mRNA level in *Dnmt1^-/- ^*MEFs (C), and H3K27me3 up in *Dnmt1^-/- ^*MEFs (D). **P <0.005 by Wilcoxon rank sum test relative to all genes. **(E) **qRT-PCR quantifying expression of the indicated genes in *Dnmt1^+/+ ^*MEFs treated with vector control or 5-aza-dC. Expression is normalized to *Actb *and expressed relative to vector control. Error bars represent ±SEM of three replicates. 5-aza-dC, 5-aza-2-deoxycytidine; DMSO, dimethyl sulfoxide; IgG, immunoglobulin G.

To validate our findings using *Dnmt1^-/- ^*MEFs, we used an independent method to induce DNA hypomethylation. We cultured *Dnmt1^+/+ ^*MEFs in the presence of a small molecule inhibitor of DNA methylation, 5-aza-2′-deoxycytidine (5-aza-dC), for 72 hours. This caused >50% loss of 5mC as measured globally by HPLC (Figure 6A, P <0.001 by t-test). ChIP-qPCR revealed that, in 5-aza-dC-treated cells, H3K27me3 enrichment was reduced at promoter regions that also lose this mark in *Dnmt1^-/- ^*MEFs, whereas it was not reduced at the positive control *Chdh *promoter (Figure 6B). 5-aza-dC treatment also resulted in increased H3K27me3 at selected regions that gain H3K27me3 in *Dnmt1^-/- ^*MEFs (Figure S6 in Additional file 1). It should be noted that the observed changes in H3K27me3 occupancy upon 5-aza-dC treatment were smaller in magnitude than those observed in *Dnmt1^-/- ^*MEFs, perhaps owing to the differences in the extent of DNA hypomethylation observed using these two methods and the short time period employed in the 5-aza-dC experiment. In order to examine gene expression changes induced by 5-aza-dC treatment of *Dnmt1^+/+ ^*MEFs, we performed expression microarrays. We examined the expression of genes that were associated with both decreased promoter H3K27me3 (H3K27me3 down genes) and increased expression (>1.5 fold) in *Dnmt1^-/- ^*MEFs. Importantly, these genes were also associated with increased expression upon demethylation of *Dnmt1^+/+ ^*MEFs with 5-aza-dC (Figure 6C). Of note, we have already shown that these genes are associated with low levels of promoter DNA methylation in *Dnmt1^+/+ ^*MEFs (Figure 2). We performed RT-qPCR in *Dnmt1^+/+ ^*MEFs treated with 5-aza-dC to validate the upregulation of these genes that was observed by microarray (Figure 6E). We also examined the expression of genes that were within 100 kb windows of the genome that show increased H3K27me3 in *Dnmt1^-/- ^*MEFs, which we showed were on average downregulated in *Dnmt1^-/- ^*MEFs (Figure S5A in Additional file 1). Importantly, upon treatment of *Dnmt1^+/+ ^*MEFs with 5-aza-dC, these genes were also downregulated on average (Figure 6D). We showed that upon 5-aza-dC treatment, the intragenic regions of the *Pcdhb18 *and *Pcdhb20 *genes were associated with increased H3K27me3 (Figure S6A in Additional file 1), and these genes were downregulated at the mRNA level (Figure S6B in Additional file 1). Importantly, this experiment demonstrated that inducing DNA hypomethylation using an independent experimental method leads to H3K27me3 redistribution and gene expression changes that are consistent with those observed in *Dnmt1^-/- ^*MEFs.

To further validate our observations we used a third independent experimental method to induce DNA hypomethylation. We generated *Dnmt1^+/+ ^*MEFs that stably expressed a short hairpin RNA against *Dnmt1 *(sh-Dnmt1 cells) [47]. We generated three independent cell lines expressing the *Dnmt1 *short hairpin RNA and three using a control plasmid. We confirmed by qRT-PCR that Dnmt1 mRNA was depleted in sh-Dnmt1 cells (Figure S7A in Additional file 1), and by HPLC that the genome was hypomethylated (Figure S7B in Additional file 1). We measured the expression of selected H3K27me3 down and H3K27me3 up genes in sh-Dnmt1 cells. Consistent with our observations using other DNA hypomethylated systems, H3K27me3 down genes were upregulated in sh-Dnmt1 cells (Figure S7C in Additional file 1) and H3K27me3 up genes were downregulated (Figure S7D in Additional file 1). This experiment showed that our observations made using *Dnmt1^-/- ^*MEFs were consistent across multiple methods for inducing DNA methylation loss of cells in culture.

To look for an effect of DNA hypomethylation on Polycomb target gene expression *in vivo*, we examined the expression of an H3K27me3 gene set in mouse embryos homozygous for a point mutation in *Dnmt1 *(referred to as *Dnmt1^PM ^*allele) [48]. This point mutation causes instability of the Dnmt1 protein and therefore results in DNA hypomethylation and embryonic lethality when homozygous [48]. We analyzed gene expression in *Dnmt1^+/PM ^*and *Dnmt1^PM/PM ^*embryos at 7.5 dpc (days post coitum), as at later stages severe developmental defects become apparent in homozygotes (Figure S8A in Additional file 1) [48]. We observed modest but consistent increases in expression of the tested Polycomb target genes in *Dnmt1^PM/PM ^*embryos at 7.5 dpc, although variation between embryos was large at this developmental stage (Figure S8B in Additional file 1). This suggests that this mechanism may be operating *in vivo *during mammalian embryonic development.

## Discussion

We have uncovered an unexpected link between DNA methylation and PRC2-mediated gene repression. An intact DNA methylome is required to restrict PRC2-function to its normal gene targets. Where DNA methylation levels are reduced in somatic cells, either genetically or by pharmacological inhibition, many Polycomb target genes lose their association with PRC2 and H3K27me3, and become ectopically expressed.

**Figure 7:**
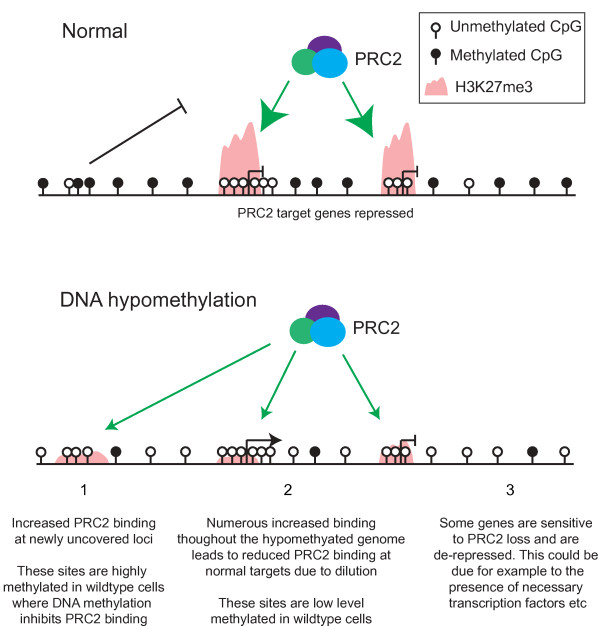
**A model for de-repression of Polycomb target genes upon loss of DNA methylation**. In normal cells, PRC2 is targeted to chromatin in a process that involves unmethylated stretches of DNA, such as CpG islands (PRC2 targeting is indicated by green arrows). DNA methylation has a negative effect on PRC2 binding to chromatin and so constrains PRC2 targeting. When global DNA methylation levels are reduced, PRC2 binding and H3K27me3 increase at numerous additional genomic loci leading to a dilution of available PRC2 from its normal targets. Note that only loci that are somehow permissive to PRC2 binding show increased H3K27me3 in hypomethylated cells. Reduced PRC2 binding to its normal target promoters results in loss of transcriptional repression. PRC2, Polycomb Repressive Complex 2.

How Polycomb complexes are targeted to specific regions of the mammalian genome is a major question [44]. Here, we followed multiple lines of evidence that implicated DNA methylation as a player in this process. In severely DNA hypomethylated mouse somatic cells, we found that H3K27me3 was redistributed in a manner that reflected the DNA methylation pattern in wild-type cells. Increased H3K27me3 occurred at many regions of the genome that are normally highly DNA methylated, consistent with the idea that DNA methylation is capable of attenuating PRC2 binding in certain genomic contexts. This finding is consistent with recently published studies that investigate patterns of H3K27me3 in hypomethylated mouse ES cells, but also suggests that this may be a function of the DNA methylome in lineage committed, in addition to pluripotent, cell types [32,37]. Importantly, we also observed decreased H3K27me3 at a large number of normal Polycomb targets that are associated with low-level DNA methylation in wild-type cells. We suggest the following model to account for both features of H3K27me3 redistribution in hypomethylated cells (Figure 7). Upon hypomethylation, DNA methylation-mediated repression of PRC2 binding is lost, allowing increased PRC2 binding and H3K27me3 modification at inappropriate genomic loci. These regions are associated with high levels of DNA methylation in wild-type cells where DNA methylation inhibits PRC2 binding. We suggest that this leads to a dilution of PRC2 away from its normal targets (Figure 7). This idea is supported by recent work using hypomethylated ES cells, where decreased H3K27me3 was observed at regions that are marked by discrete peaks of H3K27me3 in wild-type cells, and new domains of H3K27me3 are formed at ectopic locations [37]. This model makes the assumption that the amount of the PRC2 complex is limiting in these cells. We speculate that the cell must maintain appropriate levels of this complex to avoid the aberrant repression of transcription at non-target loci. This model explains the general loss of H3K27me3 that we observed from its normal target promoters, and the strong association between DNA methylation patterns and H3K27me3 changes upon hypomethylation. Further work will be required to test this hypothesis and to further define the mechanism behind the H3K27me3 redistribution observed upon loss of DNA methylation, and its consequences for gene regulation [49].

The molecular mechanism behind DNA methylation-mediated attenuation of PRC2-chromatin interactions is not yet known. *In vitro *experiments using reconstituted PRC2 and chromatin suggest that one or more PRC2 components are capable of directly differentiating CpG from 5mCpG [39]. PRC2 is thought to bind to chromatin through several DNA and histone interactions [2]. DNA methylation may be interpreted by one or more individual subunits of PRC2, or by the complex as a whole in response to a structural change of chromatin induced by DNA methylation. Several PRC2 components contain zinc finger domains that could potentially mediate this effect, as the DNA binding of certain zinc finger domains, such as CXXC domains, are directly inhibited by DNA methylation [2,19,20].

We did not observe increased H3K27me3 in hypomethylated cells at all regions of the genome that are highly DNA methylated in normal cells. This would be expected to occur if the DNA methylation pattern was the sole determinant of H3K27me3 targeting. Rather, this observation is further proof that DNA methylation is merely one of multiple signals that the cell uses to correctly position the H3K27me3 mark in the genome. This is consistent with other studies demonstrating the importance of sequence-specific DNA-binding proteins [44], transcription [31], non-coding RNA [50] and other chromatin features [51] in the targeting of H3K27me3. The final distribution of H3K27me3 is therefore likely the result of the concerted action of these multiple mechanisms. Future studies, taking into account DNA sequence, RNA, and epigenetic modulators of PRC2-binding will help to advance our understanding of this complex problem.

Importantly, we observed that loss of promoter H3K27me3 in DNA hypomethylated somatic cells was associated with transcriptional activation, consistent with a loss of Polycomb-mediated repression. This suggests that an intact DNA methylome is indirectly required for the normal repression of PRC2-target genes. This unexpected finding significantly widens the scope for genes that require DNA methylation for their repression. Owing to their low level of promoter DNA methylation, these genes would not normally be associated with repression that involves DNA methylation. Many of these genes are classic Polycomb targets, such as the *Hox *genes, and have known functions in embryonic development and cell-fate specification. In fact, *Hox *gene regulation has been previously linked to DNA methylation, but the underlying role of DNA methylation in this process has remained unclear. Firstly, mice with hypomorphic mutations in the DNA methyltransferase gene *Dnmt3b *display homeotic transformations of the posterior axis, concomitant with de-repression of certain *HoxA *genes [14]. Secondly, loss of the Lsh protein leads to DNA hypomethylation and mis-expression of certain *Hox *genes in mouse embryos and cell lines [52]. The findings presented in our study provide an alternative explanation for *Hox *gene de-repression upon hypomethylation, even when the gene of interest is not associated with a highly DNA methylated promoter. It should be noted that our knowledge of DNA methylation patterns within *Hox *clusters is incomplete. *Hox *cluster DNA methylation appears to vary in a complex tissue- and cluster-specific manner, but the CpG-rich regions associated with *Hox *gene promoters appear to remain in an unmethylated state in most tissues studied [3-6]. Indeed, in our study, we observed that the CpG-rich promoters of the *Hox *genes studied contained low levels of DNA methylation in MEFs, suggesting that their de-repression was not caused by loss of promoter DNA methylation. However, at this point we cannot completely exclude a role for promoter DNA methylation in the repression of these genes. We also note that *Hox *gene expression patterns in fibroblasts are dependent on their anatomic location, and that these patterns are maintained when fibroblasts are grown in culture [53]. Importantly, we have shown that DNA hypomethylation induced in *Dnmt1^+/+ ^*MEFs results in de-repression of *Hox *genes. This experiment compares the same starting population of cells, excluding the possibility that the observed gene expression differences are due to differential anatomic origin of the samples. However, we note that we cannot formally exclude the possibility that the severe mis-expression of *Hox *genes observed in *Dnmt1^-/- ^*MEFs was further enhanced by a potential differential anatomic origin of these cells. Nevertheless, our data demonstrate that loss of DNA methylation by itself is sufficient to induce de-repression of *Hox *genes.

Our observations could also offer an explanation for the enigmatic de-repression of PRC2-target developmental genes in cells of patients with immunodeficiency-centromeric instability-facial abnormalities (ICF) syndrome, a disorder frequently caused by mutation in *DNMT3B *[54]. A subset of PRC2-target genes that are associated with CpG-rich DNA methylation-free promoters were observed to be upregulated in cells of patients with ICF syndrome relative to controls [54]. It is feasible that *DNMT3B*-mutation induced DNA hypomethylation elsewhere in the genome drives reduction in PRC2 and H3K27me3 occupancy at these gene promoters, resulting in leaky repression. Our observations may also influence our understanding of the phenotypes of Dnmt1 loss in embryonic development and in multipotent stem cell populations, such as hematopoietic stem cells, where the phenotypes observed are compatible with mis-regulation of cell-fate specifying genes [41,55-57].

The findings presented in this study, along with others, demonstrate that DNA methylation is a major factor in determining PRC2 targeting in mouse cells. Different aspects of the DNA methylome appear to have been lost during the evolution of certain branches of organisms [58,59]. Some, such as the fruit fly *Drosophila melanogaster*, have lost CpG methylation entirely [59] and, in this organism, DNA-binding transcription factors play an important role in Polycomb targeting by binding to sequence elements called Polycomb response elements (PREs) [44]. The search for PREs in mammals so far has yielded few results [44]. It is interesting to speculate that without the restrictive activity of DNA methylation, *Drosophila *may rely more heavily on a PRE approach for Polycomb targeting. In support of this speculation, mapping of Polycomb components by ChIP in *Drosophila *has shown that, with the exception of Pc, which binds H3K27me3, Polycomb components are bound in punctate regions at PREs [44]. This is in contrast to mammals where PRC2 components are bound in more broad domains that are better associated with H3K27me3 domains [44]. We speculate that these differences could have arisen as a need to restrict PRC2 binding in *Drosophila *in the absence of CpG methylation.

Both DNA methylation and H3K27me3 patterns are dynamic during cell differentiation [3-5,7,45] and it remains to be investigated whether DNA methylation dynamics play a direct role in determining changes in H3K27me3 patterns upon lineage commitment. In this respect, loss of DNA methylation from specific loci during ES cell differentiation has been correlated with increased H3K27me3 [7]. A similar hypothesis could be addressed in various cancers, where dramatic alterations in DNA methylation are often observed (for example, [60]). Frequent observations in cancers are DNA hypomethylation of large genomic domains and hypermethylation of CpG islands. It will be intriguing to investigate the effect of DNA methylation redistribution on PRC2 targeting in cancer cells, and its effect on gene expression. Indeed, new domains of H3K27me3 have been observed in breast cancer cell lines in regions that become DNA hypomethylated [35]. Also, loss of H3K27me3 has been observed at promoters that become DNA hypermethylated in a prostate cancer cell line, consistent with the idea that DNA methylation changes can drive alteration of PRC2-chromatin interactions in cancer [34].

## Conclusions

We suggest that the DNA methylome has an unexpected role in the repression of Polycomb target genes, as it is essential for appropriate targeting of PRC2 and the H3K27me3 histone modification in mouse somatic cells. These findings significantly advance our understanding of how DNA methylation influences chromatin states and highlights the diverse ways that this epigenetic mark contributes to genome regulation.

## Materials and methods

### Cell lines and mouse strains

*Dnmt1^+/+ ^*and *Dnmt1^-/- ^*MEFs were a gift from Howard Cedar and their derivation and culture conditions have been previously described [12]. For treatment of *Dnmt1^+/+ ^*MEFs with 5-aza-dC, dimethyl sulfoxide vehicle control or 0.5 µM 5-aza-dC (Sigma-Aldrich, St Louis, MO, USA) was added to the culture media for 72 h, and the media was changed daily with fresh drug added. Stable knockdown of *Dnmt1 *was achieved by lentiviral transduction of a vector containing a short hairpin RNA against *Dnmt1 *[47] in triplicate into *Dnmt1^+/+ ^*MEFs, followed by selection using Blasticidin (Invitrogen, Life Technologies, Carlsbad, CA, USA). The *Dnmt1^PM ^*allele has been previously described as the *MommeD2 *allele [48].

### Chromatin-immunoprecipitation analyses

Detailed protocols are supplied for native and cross-linked ChIP in Text S1 in Additional file 1. Per immunoprecipitation, we used 10 µg of the following antibodies: αH3K27me3 (Merck-Millipore, Billerica, MA, USA; cat: 07-449); αH3K4me3 (Merck-Millipore; cat: 07-473); non-specific rabbit IgG (Santa Cruz Biotechnology Inc, Dallas, TX, USA; cat: sc-2027); αEzh2 (Merck-Millipore; cat: 17-662); non-specific mouse IgG (Santa Cruz Biotechnology Inc; cat: sc-2025). Region-specific primers used for ChIP-qPCR are shown in Table S1 in Additional file 1. For ChIP-chip, input and immunoprecipitated DNA from three replicates was amplified using the WGA2 whole genome amplification kit (Sigma-Aldrich). For promoter arrays, amplified DNA was labeled and hybridized to Mouse ChIP-chip 3x720K RefSeq Promoter Arrays (Roche NimbleGen Inc, Madison, WI, USA) by the Microarray Core Facility of the VU University Medical Center, Amsterdam, The Netherlands. Data were extracted using NimbleScan v2.5 (Roche NimbleGen). ChIP-seq libraries were sequenced using single-end 35 bp sequencing on an Illumina Genome Analyser II (performed by Ambry Genetics, Aliso Viejo, CA, USA). The number of reads that were successfully mapped to the genome and used in the analysis is shown in Table S2 in Additional file 1.

### Western blots

Detailed protocols are supplied for protein extract production and western blot in Text S1 in Additional file 1. The following primary antibodies were used at the stated dilutions: αH3K27me3 (Merck-Millipore; cat: 07-449; 1:1000); αH3K4me3 (Merck-Millipore; cat: 07-473; 1:1000); αH3 (Abcam, Cambridge, UK; cat: ab1791; 1:1000); αEzh2 (Merck-Millipore; cat: 17-662; 1:1000); αSuz12 (Cell Signaling Technology, Beverly, MA, USA; cat: D39F6#3737S; 1:1000); αEed (Merck-Millipore; cat: 05-1320; 1:5000); αActb (Abcam; cat: ab3280; 1:5000).

### DNA methylation analyses

Quantitation of methyl-cytosine in genomic DNA by HPLC was performed as previously described [61] with the following alterations. To improve peak resolution, the column was chilled to 8°C. Deoxyribonucleotides were detected using a Dionex 3000 multiple wavelength detector at their extinction maxima: dCMP (deoxycytosine monophosphate), 276 nm; 5mdCMP (5'-methyl-deoxycytosine monophosphate), 282 nm. Quantifications were calculated from the area under each peak using the respective extinction coefficients (dCMP, 8.86 × 10^3^; 5mdCMP, 9.0 × 10^3^). RRBS was performed by BaseClear (Leiden, The Netherlands) as provided by the 'Epiquest Genome-wide Basic Service'. HELP-tag-seq was performed as previously described [62-64] using a custom mouse *MspI *library. Bisulfite sequencing was performed as described in Text S1 in Additional file 1. Primers used for bisulfite sequencing are shown in Table S1 in Additional file 1.

### Gene expression assays

Total RNA was isolated from cells and tissues in triplicate using Trizol (Life Technologies, Invitrogen) according to the manufacturer's instructions. RNA integrity was determined by agarose gel electrophoresis and/or Bioanalyser RNA6000 Nano chip (Agilent, Santa Clara, CA, USA). Only RNA with an RNA integrity score >9 was used. qRT-PCR was performed as described in Text S1 in Additional file 1. qRT-PCR primers are shown in Table S1 in Additional file 1. For Illumina BeadChip expression microarrays, RNA was amplified using a TotalPrep RNA amplification kit (Life Technologies, Ambion) and analyzed on Illumina (San Diego, CA, USA) MouseWG-6 v2.0 Expression BeadChips (hybridization and scanning performed by the Genetics Core at the Wellcome Trust Clinical Research Facility, Edinburgh, UK). For mRNA-seq, libraries were generated using the Illumina strand-specificity protocol [65], and sequenced using 50 bp single-end sequencing on an Illumina HiSeq2000 sequencer (performed by Ambry Genetics).

### Data analysis

For the methods used in the normalization and analysis of data from ChIP-chip, ChIP-seq, RRBS, HELP-tag-seq, Illumina BeadChip expression microarray, and mRNA-seq see Text S1 in Additional file 1.

### Data access

Raw and processed data are deposited into the Gene Expression Omnibus (GEO accession number: GSE44278).

## Abbreviations

5-aza-dC: 5-aza-2-deoxycytidine; bp: base pair; ChIP: chromatin-immunoprecipitation; Dnmt1: DNA methyltransferase 1; ES: embryonic stem; H3K27me3: histone H3 lysine 27 trimethylation; H3K4me3: histone H3 lysine 4 trimethylation; HELP-tag-seq: HpaII tiny fragment enrichment by ligation-mediated PCR followed by tag sequencing; HPLC: high performance liquid chromatography; IgG: immunoglobulin G; mC: methyl-cytosine; MEF: mouse embryonic fibroblasts; PRC1: Polycomb Repressive Complex 1; PRC2: Polycomb Repressive Complex 2; PRE: Polycomb response element; RRBS: reduced-representation bisulfite sequencing.

## Competing interests

The authors declare that they have no competing interests.

## Authors' contributions

JPR and RRM conceived and designed the experiments. JPR, SMP, NAY, JR, HM, MS, DR, DSD and BHR performed the experiments. JPR, CEN and JGP analyzed the data. EW, JMG, IRA and WAB contributed reagents, materials or analysis tools. JPR and RRM wrote the paper. All authors read and approved the final manuscript.

## Supplementary Material

Additional file 1 **A PDF document containing supplemental files for this paper**. Included are eight supplemental figures (Figures S1 to S8), supplemental materials and methods (Text S1), and two supplemental tables (Tables S1 and S2). Click here for file

Additional file 2 An Excel spread sheet (XLS) showing normalized average enrichments and *P*-values observed at gene promoter regions by ChIP-chip for H3K27me3 and H3K4me3 in *Dnmt1^+/+ ^*and *Dnmt1^-/- ^*MEFs. Click here for file

Additional file 3 An Excel spread sheet (XLS) showing genomic windows (100 kb) that show differential H3K27me3 by ChIP-seq in *Dnmt1^+/+ ^*and *Dnmt1^-/- ^*MEFs, along with the number of H3K27me3 reads overlapping each window in each sample and their odds ratios. Click here for file

Additional file 4 An Excel spread sheet (XLSX - compressed in ZIP) showing genomic windows (1 kb) that show differential H3K27me3 by ChIP-seq in *Dnmt1^+/+ ^*and *Dnmt1^-/- ^*MEFs, along with the number of H3K27me3 reads overlapping each window in each sample and their odds ratios. Click here for file
