# Supplementary material for: Redistribution of H3K27me3 upon DNA hypomethylation results in de-repression of Polycomb target genes
Source: Genome Biol. 2013 Mar 25;14(3):R25. doi: 10.1186/gb-2013-14-3-r25 (PMC4053768; doi:10.1186/gb-2013-14-3-r25)
Supplement: Additional file 1 — A PDF document containing supplemental files for this paper. Included are eight supplemental figures (Figures S1 to S8), supplemental materials and methods (Text S1), and two supplemental tables (Tables S1 and S2). [file gb-2013-14-3-r25-S1.PDF]

Additional File 1

Supplemental Information for Reddington et al. (2013), 'Redistribution of H3K27me3 upon DNA hypomethylation results in de-repression of Polycomb-target genes' in Genome Biology.

This file contains:

- 8 Supplemental Figures (Figures S1 to S8)
- Text S1 – extended materials and methods
- Tables S1 and S2

Figure S1

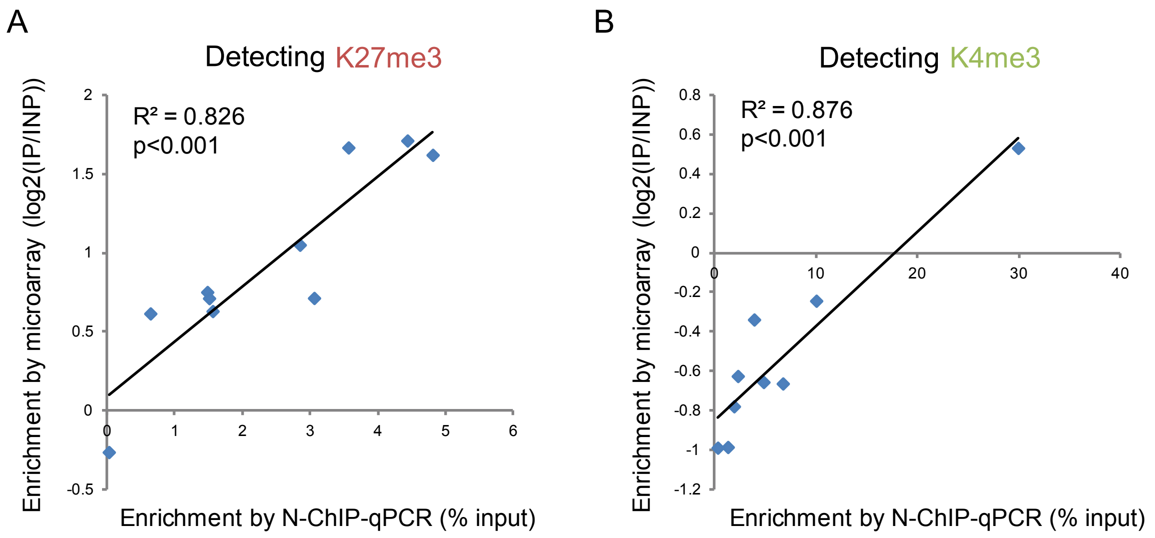

Figure S1. Validation of the promoter ChIP-chip approach using ChIP-qPCR. (A and B) Scatter plots showing the values obtained for H3K27me3 (A) and H3K4me3 (B) for 9 promoter regions comparing promoter ChIP-chip and ChIP-qPCR. ChIP-chip enrichment is displayed as log2(IP/INP) and ChIP-qPCR is displayed as % input. A best fit line was fitted by regression and the R2 value (Pearson correlation) is shown.

Figure S2

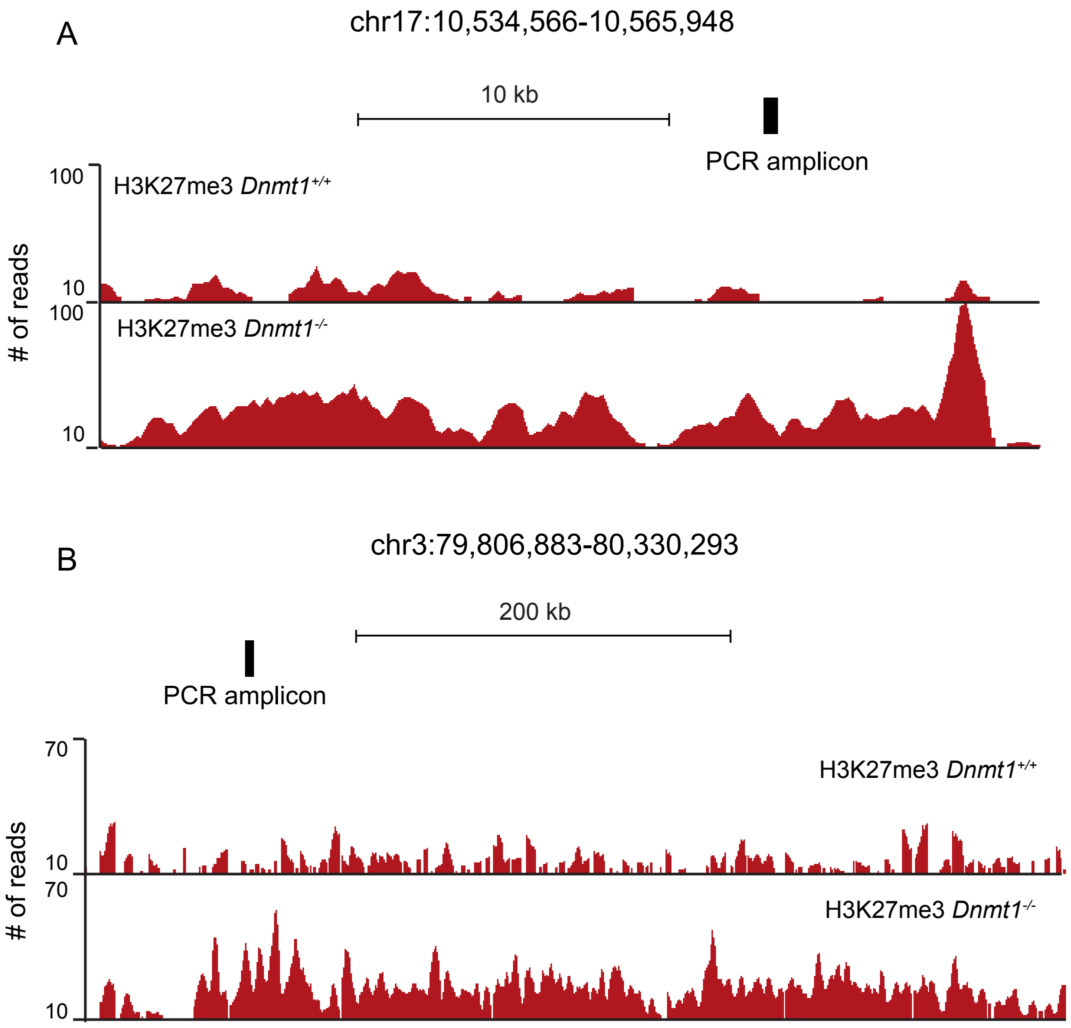

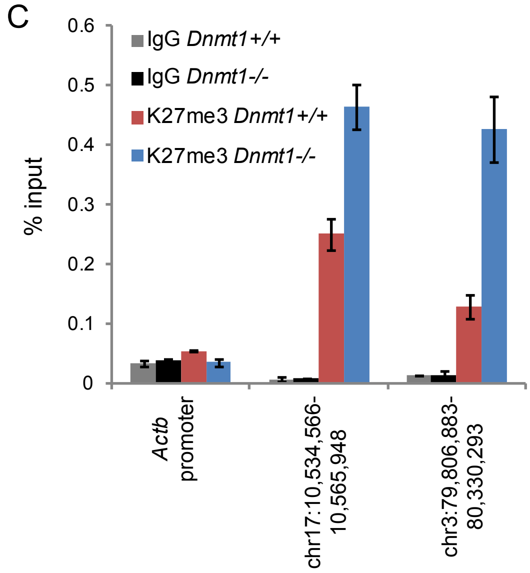

Figure S2. H3K27me3 is increased in certain intergenic regions upon loss of DNA methylation. (A and B) Browser shots from the UCSC genome browser displaying ChIP-seq data for H3K27me3 in Dnmt1+/+ and Dnmt1-/- MEFs for two intergenic regions that were defined as H3K27me3 up by genomic window analysis. The chromosomal coordinates and genomic scale are shown above each image. The position of a PCR amplicon used to validate changes is shown as a black rectangle. (C) ChIP-qPCR for H3K27me3 in Dnmt1+/+ and Dnmt1-/- MEFs using primers for the regions shown in A and B. Error bars indicate S.E.M. for 2 experiments.

Figure S3

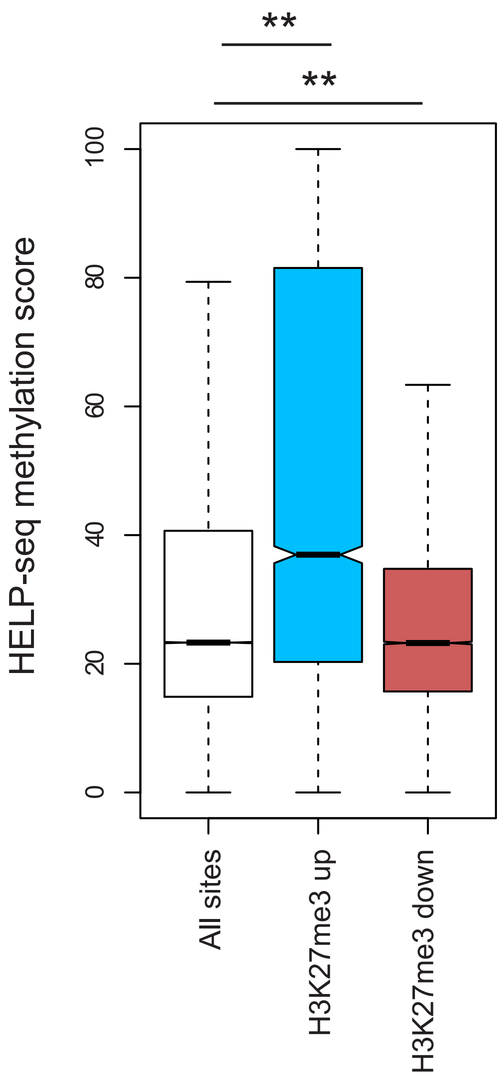

Figure S3. H3K27me3 changes upon DNA hypomethylation are associated with DNA methylation levels in wildtype cells as measured by HELP-tag-seq. Boxplot showing the CpG methylation score in Dnmt1+/+ MEFs as calculated by HELP-tag-seq for sites within regions defined as H3K27me3 up and down by ChIP-seq. ** $p<0.0001$ by Wilcoxon rank sum test between the indicated groups.

Figure S4

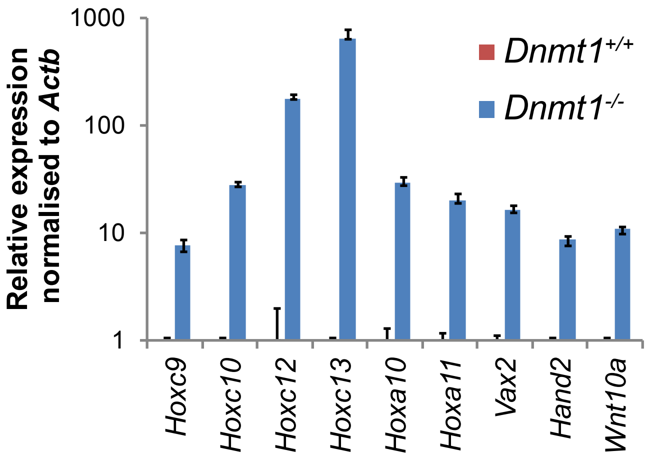

Figure S4. Validation of mRNA upregulation of Polycomb target genes in Dnmt1-/- MEFs. qRT-PCR quantifying expression of the indicated genes in Dnmt1+/+ and Dnmt1-/- MEFs. Expression is normalised to Actb and expressed relative to Dnmt1+/+ MEFs. Error bars represent S.E.M. of 3 replicates.

Figure S5

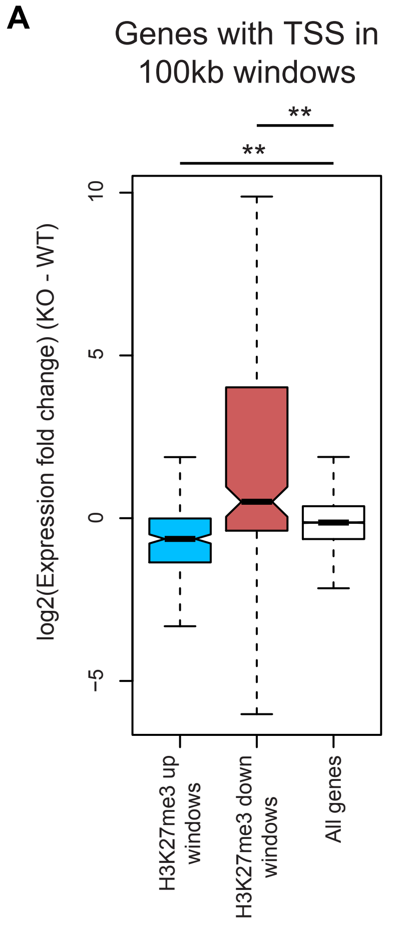

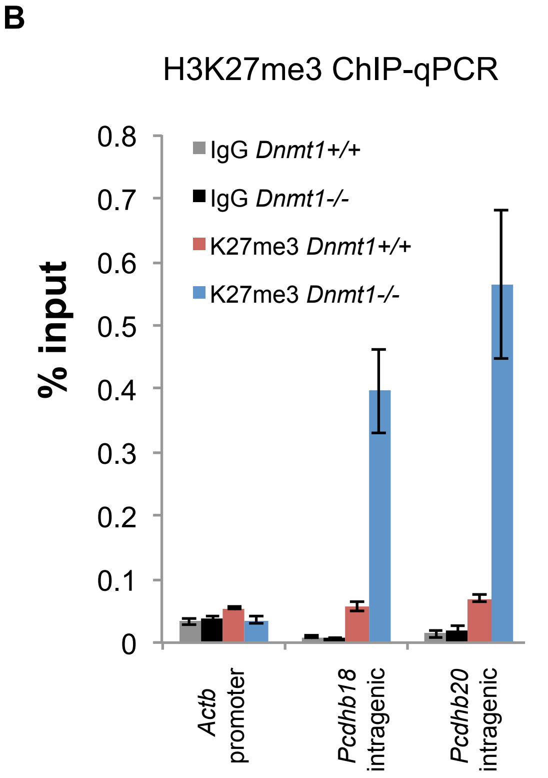

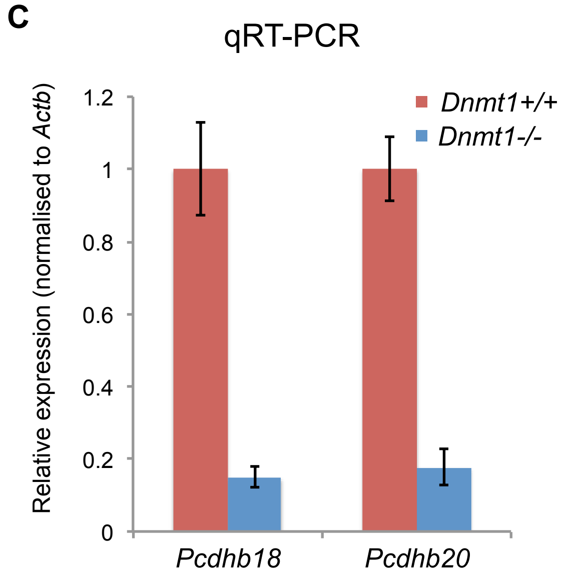

Figure S5. H3K27me3 redistribution in DNA hypomethylated cells is associated with altered gene expression. (A) Boxplot showing the relative expression of genes that have a transcription start site within large (100kb) windows found to contain increased or decreased H3K27me3 in Dnmt1-/- relative to Dnmt1+/+ MEFs. The relative expression between Dnmt1-/- relative to Dnmt1+/+ MEFs is plotted as calculated by mRNA-seq. ** $p<0.0001$ by Wilcoxon rank sum test between the indicated groups. (B) ChIP-qPCR for H3K27me3 in Dnmt1+/+ and Dnmt1-/- MEFs using primers for intragenic regions of the indicated genes. Enrichment for each IP is expressed as percentage of DNA in input. Non-specific IgG IP and the Actb promoter are shown as negative controls. Error bars indicate +/- S.E.M. of 2 experiments. (C) qRT-PCR for the indicated genes in Dnmt1+/+ and Dnmt1-/- MEFs. Error bars indicate +/- S.E.M for 3 replicates.

Figure S6

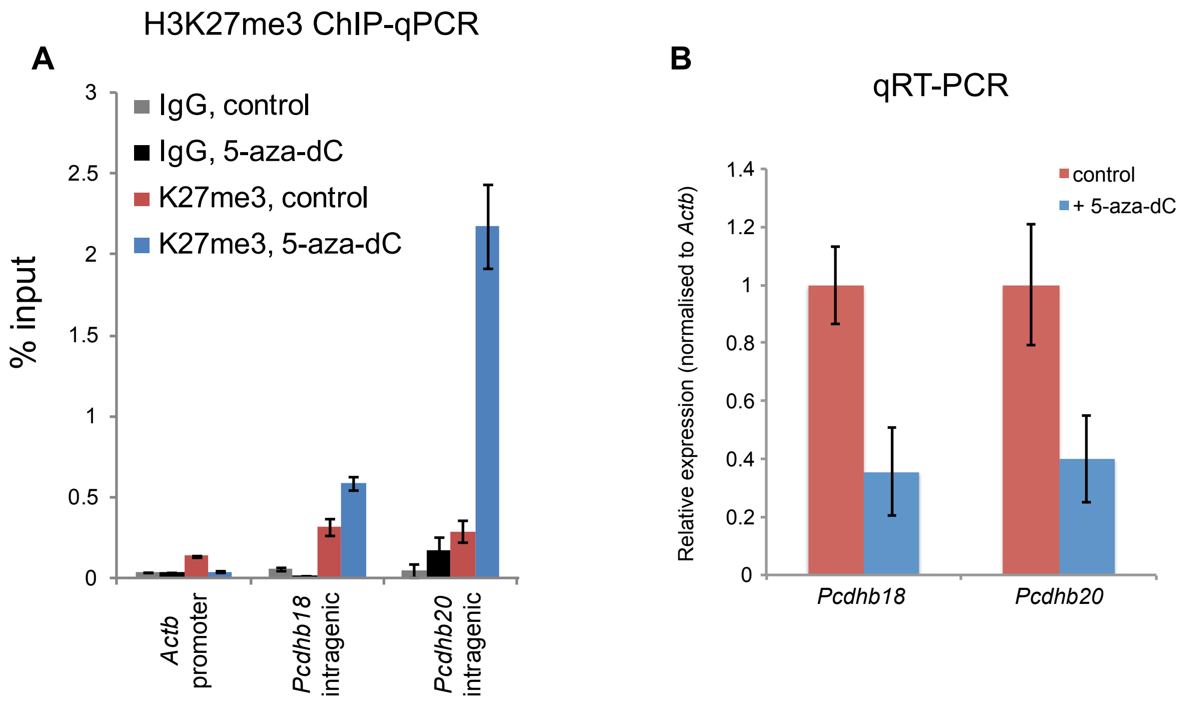

Figure S6. Demethylation using 5-aza-dC results in increased H3K27me3 at regions shown to gain H3K27me3 in Dnmt1-/- MEFs. (A) ChIP-qPCR for H3K27me3 in Dnmt1+/+ MEFs treated with either vector control or 5-aza-dC using primers for intragenic regions of the indicated genes. Enrichment for each IP is expressed as percentage of DNA in input. Non-specific IgG IP and the Actb promoter are shown as negative controls. Error bars indicate +/- S.E.M. of 2 experiments. (B) qRT-PCR for the indicated genes in Dnmt1+/+ MEFs treated with either vector control or 5-aza-dC. Error bars indicate +/- S.E.M for 3 replicates.

Figure S7

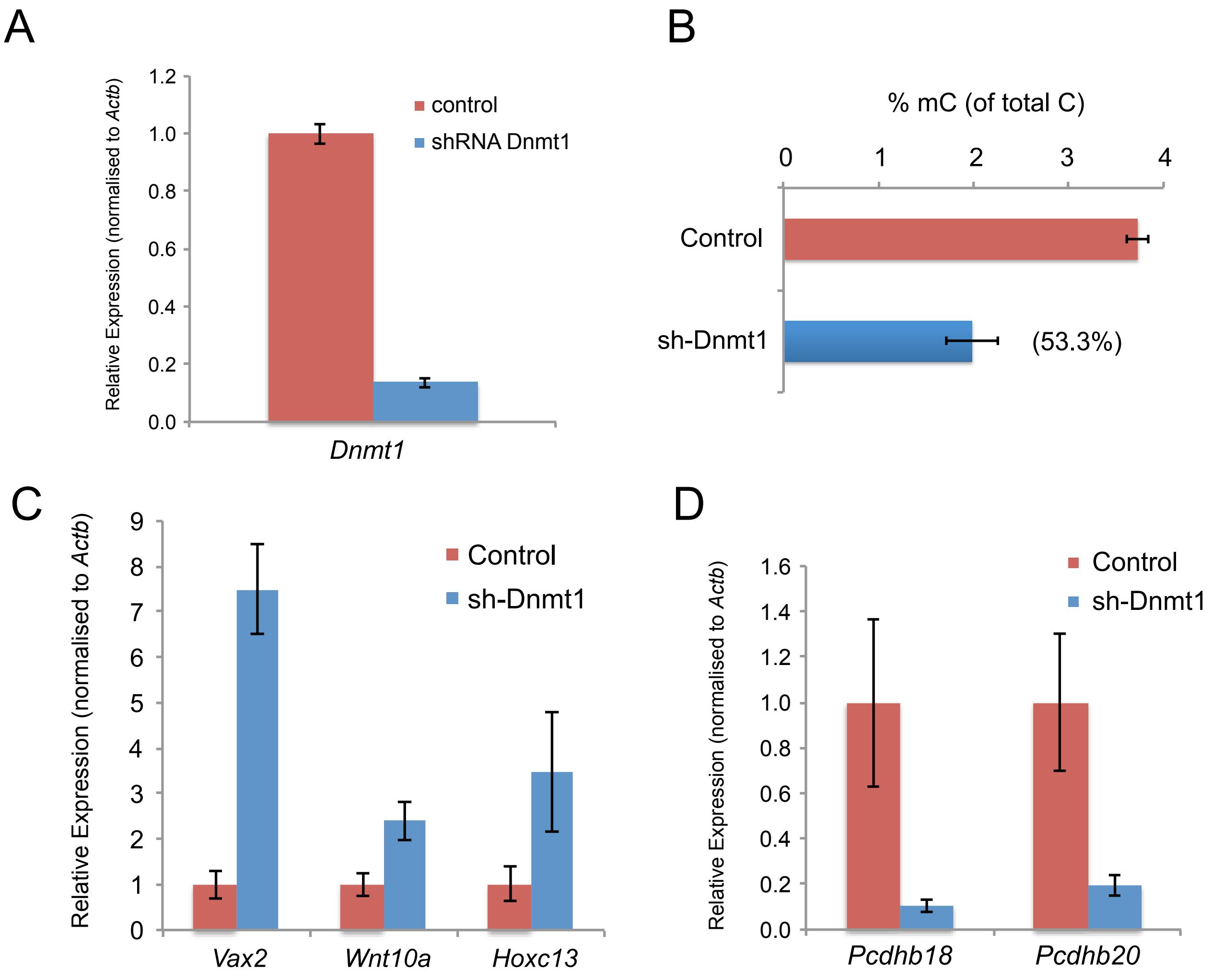

Figure S7. Stable knockdown of Dnmt1 results in DNA hypomethylation and de-repression of polycomb-target genes. (A) Dnmt1+/+ MEFs stably expressing a short hairpin RNA against Dnmt1 (sh-Dnmt1 cells) show reduced expression of Dnmt1 mRNA by qRT-PCR compared to controls. Error bars indicate +/- S.E.M of 3 replicates. (B) HPLC shows that global levels of methyl-cytosine in DNA are reduced in sh-Dnmt1 cells. Error bars indicate +/- S.E.M of 3 replicates. (C) sh-Dnmt1 cells show increased expression of polycomb-target genes relative to controls by qRT-PCR. Error bars indicate +/- S.E.M of 3 replicates. (D) sh-Dnmt1 cells show reduced expression of H3K27me3 up genes relative to controls by qRT-PCR. Error bars indicate +/- S.E.M of 3 replicates.

Figure S8

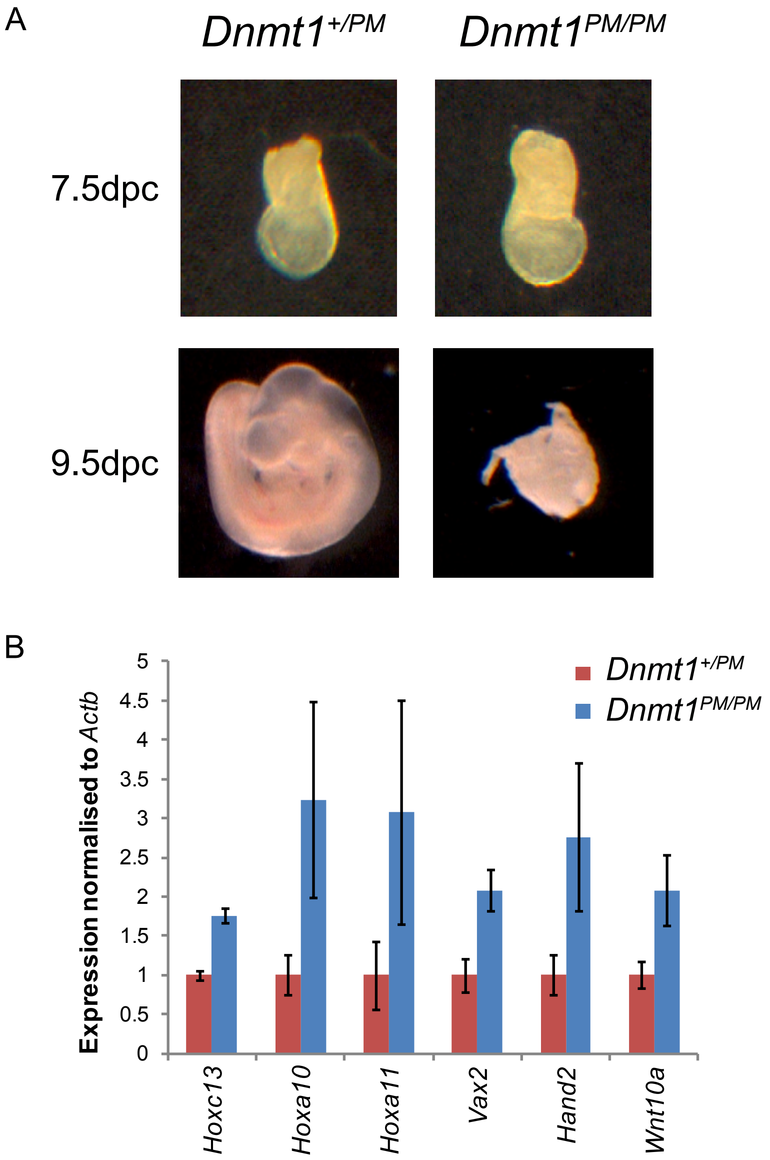

Figure S8. Upregulation of Polycomb target genes in Dnmt1-mutant mouse embryos. (A) Representative images of 7.5dpc and 9.5dpc mouse embryos either heterozygous or homozygous for a point mutant of Dnmt1 (Dnmt1PM - previously called MommeD2) . (B) qRT-PCR quantifying expression of the indicated genes in RNA from 7.5dpc Dnmt1+/PM and Dnmt1PM/PM mouse embryos. Expression is normalised to Actb and displayed relative to Dnmt1+/PM embryos. Error bars represent +/- S.E.M. for 3 biological replicates.

Text S1: Supplemental Materials and Methods

Native chromatin immunoprecipitation (N-ChIP)

N-ChIP was performed as described in [1] with the following adaptations. Protein-A coated Dynabeads (Invitrogen) were used throughout. Chromatin was digested with 25U of Mnase (micrococcal nuclease)(Worthington) for exactly 10min at room temperature. Following immunoprecipitation beads were washed three times for 10 min at 4°C in N-ChIP wash buffer (150mM NaCl; 10mM Tris pH8; 2mM EDTA; 1% NP40; 1% sodium deoxycholate w/v). Controls for N-ChIP experiments included a species matched IgG to control for background, negative and positive genomic regions to show specificity, and the use of input DNA to control for technical variations between cell lines or treatments. Antibodies were tested by western blot for recognition of histone-sized proteins only and by specific enrichment at positive control regions by N-ChIP. Enrichments were measured by qPCR.

Cross-linked chromatin immunoprecipitation (X-ChIP)

X-ChIP was performed as described in [1] with the following adaptations. M280 Dynabeads (Invitrogen) coated with sheep anti-mouse IgG were used throughout. Following immunoprecipitation beads were washed once in PBS-RIPA (1xPBS; 1% NP40 v/v; 0.5% sodium deoxycholate w/v; 0.1% SDS w/v; complete EDTA-free protease inhibitor tablet (Roche)), once in X-ChIP wash buffer (150mM NaCl; 10mM Tris pH8; 2mM EDTA; 1% NP40; 0.1% sodium deoxycholate w/v), and once in LiCl wash buffer (100mM Tris pH7.5; 500mM LiCl; 1% NP40; 1% sodium deoxycholate) for 10 min at 4°C each wash. Sonication was performed using a 'Soniprep 150' probe sonicator (MSE) at 6µm amplitude for 30sec on/off cycles for 5 cycles with incubation on ice between cycles. Controls for X-ChIP experiments included a species matched IgG to control for background, negative and positive genomic regions to show specificity, and the use of input DNA to control for technical variations between cell lines or treatments. Antibodies were tested by western blot for recognition of proteins of the expected size and by specific enrichment at positive control regions by X-ChIP. Enrichments were measured by qPCR.

Quantitation of ChIP DNA using qPCR

For ChIP-qPCR, 20µl reactions were performed in duplicate on a LightCycler480 (Roche) using Brilliant II QPCR Master Mix (Agilent), 0.5µl eluted DNA and region specific primers (Table S1). Serial dilutions of input DNA was used to set a standard curve. Enrichments were expressed as percentage of input.

Histone and whole-cell extracts

Whole cell extracts were prepared by lysing cells in SDS loading buffer (60mM Tris pH6.8; 10% glycerol; 2% SDS w/v; 0.01% bromophenol blue w/v; 10mM DTT). The extract was sonicated using a 'Soniprep 150' probe sonicator (MSE)(3 pulses of 30s at 10µm amplitude), centrifuged to remove debris (5min at 20k*g at room temperature) and then boiled at 95°C for 5min. For histone modifications, the input for native ChIP was used. Protein concentration of extracts was measured by methanol precipitation from SDS loading buffer followed by Bradford assay.

Western blotting

Protein was resolved by sodium dodecyl sulfate polyacrylamide gel electrophoresis (SDS-PAGE) using precast NuPAGE 4-12% gradient gels (Invitrogen). Following electrophoresis gels were transferred to PVDF membrane (BioRad) in transfer buffer (25mM Tris; 200mM glycine; 20% methanol v/v) using a GenieBlotter (Idea Scientific). Membranes were blocked with PBS (phospho-buffered saline) with 0.1% Tween20 (Sigma) and 5% non-fat dry milk proteins (Marvel)(PBST-milk) for at least 30min. Detection was performed using 'SuperSignal Western Pico Reagent' chemi-luminescence reagent (Pierce) and 'ECL Hyperfilm' (Amersham).

Bisulfite sequencing

500ng of genomic DNA was bisulfite treated using 'EZ Methylation Gold' kit (Zymo research) according to the manufacturer's instructions. Bisulfite primers were designed using the 'Bisearch' algorithm (http://bisearch.enzim.hu/) and are shown in Table S1. Products were amplified by two rounds of PCR, the second using nested primers, then resolved by agarose gel electrophoresis. A negative control reaction containing dH_20 in place of bisulfite-converted DNA was used for each set of reactions. Products were gel extracted, cloned into pGEM T Easy vector (Promega) and transformed into Library Efficiency DH5α bacteria (Invitrogen). Blue/white screening was performed using X-gal (Sigma). Plasmid DNA from individual colonies was isolated by miniprep and sequenced using SP6 sequencing primer using the dideoxy sequencing method. The sequences were imported into the 'BioEdit' program (http://www.mbio.ncsu.edu/bioedit/bioedit.html) and aligned to a reference sequence for the region, created by *in silico* bisulfite treatment, using the 'ClustalW' inbuilt program. Clones with identical sequences were removed due to the possibility of PCR bias. Clones with <95% conversion rate of C to T were excluded. The methylation status of each CpG dinucleotide from each bacterial clone was assessed by the presence or absence of C to T conversion in the PCR product, and visualised using 'QUMA' online program [2] (http://quma.cdb.riken.jp/). The overall percentage CpG methylation was calculated for each region.

Preparation of cDNA and qRT-PCR

Where possible, RNA was treated with DNase to remove any DNA contamination using 'TURBO DNase-free' (Ambion) according to manufacturer's instructions. In addition, all primers used for RT-PCR are intron-spanning to avoid amplification of genomic DNA (Table S1). Between 200ng and 5µg of total RNA was used as a template for cDNA synthesis by reverse transcription primed by oligo-dT primers (Promega) using Superscript III (Invitrogen). Negative control cDNA synthesis reactions were performed without the addition of the reverse transcriptase. qRT-PCR primers (Table S1) were designed using the 'Primer3' program (http://frodo.wi.mit.edu/primer3/) and were screened for the formation of multiple products or extensive primer-dimers. qPCR on cDNA was performed in duplicate using a LightCycler480 (Roche) and Brilliant III QPCR Master Mix (Agilent). Serial dilution of a

cDNA sample known to contain the target transcript was performed to calculate a standard curve. Relative abundance of each target in the measured samples was calculated and normalised to *Actb* as a loading control. Standard error of the mean was calculated and propagated where necessary.

Data analysis: General

The mm9 (July 2007, NCBI37) build and annotation of the mouse genome was used throughout this study. The 'R' statistical environment (http://www.R-project.org)(version 2.12.1) and a local version of the 'Galaxy' platform [3-5] were used for multiple methods in this section.

Data analysis: ChIP-chip

Data was extracted using NimbleScan v2.5 (NimbleGen). Quality control included pair-wise comparisons of data to ensure a good correlation between replicates. Dye-bias was corrected by intra-array lowess normalisation using the *limma* package [6]. Systematic variation between arrays was corrected by inter-array scale normalisation using the *limma* package [6]. In general, ChIP-chip data was expressed as the log2 ratio of immunoprecipitated DNA to input DNA (log2(IP/INP)), which was calculated for each probe. For NimbleGen promoter microarrays, genomic regions covered by the array were defined as promoters of RefSeq transcripts if the transcript transcription start site falls within the covered region. Promoters differentially marked by a given histone modification between conditions were defined as follows. The mean log2(IP/INP) for all probes in a promoter region was calculated. A two-tailed t-test was performed between the 3 replicates of each condition. The p-values were corrected for multiple testing by the Benjamini-Hochberg method [7], yielding q-values (equivalent to false discovery rate). To be defined as differentially marked between conditions, a promoter region must have a q-value < 0.05 and a difference in log2(IP/INP) > 0.5 (or < -0.5). Gene ontology (GO) enrichments were assessed using 'Babelomics 4.2' (http://babelomics.bioinfo.cipf.es/), specifically the 'FATIGO' program. This program performs a two-tailed Fisher's exact test on the GO terms from two

lists of genes that are provided by the user. As multiple hypotheses are questioned, the p-value is corrected by the false discovery rate (FDR) procedure of Benjamini and Hochberg [7], yielding a q-value. A list of promoters covered by the promoter microarray was used as background. For a background for GO analysis, we defined promoters that are marked by H3K27me3 in control cells as having a mean log2(IP/INP) > 0.7.

Data analysis: ChIP-seq

ChIP-seq reads were generated on an Illumina Genome Analyser II and mapped to the mm9 build of the mouse genome using the bowtie short read aligner [8]. Where a read mapped to a number of potential locations, only the best hit was retained (in terms of the number of mismatches and their qualities; as specified by the bowtie '–best' parameter). Approximately 36 million reads were successfully mapped for each sample (see Table S2 for exact numbers). BEDTools [9] was used to count mapped reads that overlap genomic windows. Two different window sizes were used: 1kb (200bp slide) and 100kb (20kb slide). A Fisher's exact test was applied to look for windows with differential numbers of mapped reads. For 1kb windows, those containing less than a total of 20 reads from both conditions were excluded. The p-values were corrected for multiple testing by the Benjamini-Hochberg method [7] yielding q-values. Windows were considered significantly different between conditions if their $q < 0.05$ and their odds ratio is > 3 (or < 0.33333). Pile-up of reads around H3K27me3 marked genes was performed as follows. The top 10% of promoters were selected from NimbleGen promoter ChIP-chip data ranked by mean H3K27me3 enrichment in *Dnmt1*$^{+/+}$ MEFs. The mm9 RefGene table was used to define genomic coordinates for these genes. BEDTools was used to count the number of H3K27me3 sequence reads that overlap 100bp sliding windows around and within genes. Within genes, windows were positioned with regular spacing according to proportions of gene length (e.g. at 10%, 20% of the genes length). The mean number of overlapping reads was plotted.

Data analysis: Reduced Representation Bisulfite Sequencing

BaseClear (Netherlands) performed quality control, sequence processing and mapping of sequence reads as per their 'EpiQuest Genome-wide Basic' service package. Sequence depth was determined per CpG dinucleotide as the number of reads where methylation status could be determined and CpGs with a depth less than 10 reads in either condition were excluded. Methylation status of the remaining CpGs was calculated as the percentage of methyl-cytosine in total cytosine.

Data analysis: HELP-tag-seq

Analysis of HELP-tag-seq data was performed as described in [10]. A cut-off of c=10 was used to filter sites with few tags. A more intuitive 'Methylation Score' was calculated from the angle value for each remaining CCGG site as follows. First, the angle values were manipulated to meet a range of 100 (0-100), as opposed to 90 (0-90). Then, this manipulated angle value was subtracted from 100, yielding higher values for more methylated sites.

Data analysis: Illumina BeadChip Expression Microarray

Data from Illumina BeadChip expression microarrays were processed using the *lumi* R package according to [11]. Background subtraction was performed using the 'forcePositive' option to ensure that only positive values were achieved. Data was transformed using Variance Stabilising Transformation [12] and normalised using Robust Spline Normalisation. The mean signal for each gene across replicates was calculated and used to calculate fold change between treatments.

Data analysis: mRNA-seq

Data from mRNA-seq was processed using the Tuxedo suite of programs [13]. Sequence reads were mapped to the mm9 genome using the Tophat program [13-14]. For the number of successfully mapped sequence reads see Table S2. Transcripts were assembled using the

Cufflinks program [13-14]. Transcript abundance and differential expression was measured using the Cuffdiff program [13]. Only transcripts where enough reads were achieved for quantification were included (as determined by 'status - OK'). Infinite relative expression values (where RPKM in one condition is 0) were set to a maximum log2(10). For determining a list of >2-fold upregulated genes for plotting a venn diagram, transcripts were defined by fold change and by having q<0.05 (p-value corrected for multiple testing by Benjamini Hochberg method).

Supplementary references

1. Eskeland R, Leeb M, Grimes GR, Kress C, Boyle S, et al. (2010) Ring1B compacts chromatin structure and represses gene expression independent of histone ubiquitination. Mol Cell 38: 452-464.
2. Kumaki Y, Oda M, Okano M (2008) QUMA: quantification tool for methylation analysis. Nucleic Acids Res 36: W170-175.
3. Blankenberg D, Von Kuster G, Coraor N, Ananda G, Lazarus R, et al. (2010) Galaxy: a web-based genome analysis tool for experimentalists. Curr Protoc Mol Biol Chapter 19: Unit 19 10 11-21.
4. Giardine B, Riemer C, Hardison RC, Burhans R, Elnitski L, et al. (2005) Galaxy: a platform for interactive large-scale genome analysis. Genome Res 15: 1451-1455.
5. Goecks J, Nekrutenko A, Taylor J, Team G (2010) Galaxy: a comprehensive approach for supporting accessible, reproducible, and transparent computational research in the life sciences. Genome Biology 11.
6. Smyth GK, Speed T (2003) Normalization of cDNA microarray data. Methods 31: 265-273.
7. Benjamini Y, Hochberg Y (1995) Controlling the False Discovery Rate - a Practical and Powerful Approach to Multiple Testing. Journal of the Royal Statistical Society Series B-Methodological 57: 289-300.
8. Langmead B, Trapnell C, Pop M, Salzberg SL (2009) Ultrafast and memory-efficient alignment of short DNA sequences to the human genome. Genome Biology 10: R25.
9. Quinlan AR, Hall IM (2010) BEDTools: a flexible suite of utilities for comparing genomic features. Bioinformatics 26: 841-842.
10. Suzuki M, Jing Q, Lia D, Pascual M, McLellan A, et al. (2010) Optimized design and data analysis of tag-based cytosine methylation assays. Genome Biol 11: R36.
11. Du P, Kibbe WA, Lin SM (2008) lumi: a pipeline for processing Illumina microarray. Bioinformatics 24: 1547-1548.
12. Lin SM, Du P, Huber W, Kibbe WA (2008) Model-based variance-stabilizing transformation for Illumina microarray data. Nucleic Acids Research 36.
13. Trapnell C, Roberts A, Goff L, Pertea G, Kim D, et al. (2012) Differential gene and transcript expression analysis of RNA-seq experiments with TopHat and Cufflinks. Nat Protoc 7: 562-578.
14. Trapnell C, Williams BA, Pertea G, Mortazavi A, Kwan G, et al. (2010) Transcript assembly and quantification by RNA-Seq reveals unannotated transcripts and isoform switching during cell differentiation. Nat Biotechnol 28: 511-515.

Table S1. Primer sequences used in this study

Primer pair name	**Application**	**Target**	**Sequence of forward and reverse primers (5'-3')**
**RT_Actb**	RT	*Actb*	AGAGCTATGAGCTGCCTGACG; TGTGTTGGCATAGAGGTCTTTACG
**RT_Hoxc9**	RT	*Hoxc9*	CAGCAAGCACAAAGAGGAGA; CGACGGTCCCTGGTTAAATA
**RT_Hoxc10**	RT	*Hoxc10*	GACACCTCGGATAACGAAGC; CCTCTTCTTCCTTCCGCTCT
**RT_Hoxc12**	RT	*Hoxc12*	TCAACGAGGGCAATAAGAGC; AGCTGCAACTTCGAATACGG
**RT_Hoxc13**	RT	*Hoxc13*	TACCAGCACTGGGCTCTTTC; CTCACTTCGGGCTGTAGAGG
**RT_Hoxa10**	RT	*Hoxa10*	CAGCCCCTTCAGAAAACAGT; TGTAAGGGCAGCGTTTCTTC
**RT_Hoxa11**	RT	*Hoxa11*	GTCTTCCGGCCACACTGA; GAACTCTCGCTCCAGCTCTC
**RT_Pcdhb18**	RT	*Pcdhb18*	AGCCTTGGCATCTGTGTCTT; CAGTGTTCCTGTACCGCTGA
**RT_Pcdhb20**	RT	*Pcdhb20*	GAGCTGGTTCTGGACAAAGC; GGGACCTGCACCTCATAGAA
**RT_Dnmt1**	RT	*Dnmt1*	CTCCCGAGGCCTTTACTTTC; TGCTCAGGGTACAGGGTCTC
**RT_Vax2**	RT	*Vax2*	AGTATGTGGTGGGCCGAGAG; CTGGTCCTTCTTCTGCTTGG
**RT_Hand2**	RT	*Hand2*	TCAAGGCGGAGATCAAGAAG; TGGTTTTCTTGTCGTTGCTG
**RT_Wnt10a**	RT	*Wnt10a*	CCTCACAGAGACATCCATGC; CCGTGGCATTTGCACTTAC
**ChIP_Actb**	ChIP	Promoter	GAATGTGGCTGCAAAGAGTCTAC; CTTCGCTCTCTCGTGGCTAGTA
**ChIP_Hoxc4**	ChIP	Promoter	CATCGATCCGAAATTTCCTC; TACAGCTCCTGGTGGTGATG
**ChIP_Hoxc5**	ChIP	Promoter	CGGCTTCCATCACTAACCTC; ATGCGCTCTTCCCAAATAAG
**ChIP_Hoxc8**	ChIP	Promoter	CCTATTACGACTGCCGGTTC; CGTGGTGGAAGAAGTCTTGG
**ChIP_Hoxc9**	ChIP	Promoter	TCAGTCTGGGCTCCAAAGTC; AGAGGTAGCCTCCCCAGAAC
**ChIP_Hoxc10**	ChIP	Promoter	GCTAGGTGGCGCTGTTACTC; CCAATGGGATTTGAAAATGG
**ChIP_Hoxc11**	ChIP	Promoter	GGCAGGAGAAGAGAACGATG; TGGGCAGATAGAGGTTGGAG
**ChIP_Hoxc12**	ChIP	Promoter	AAATCTGTTCCTGCCTTTCG; TCTACTCACGTGGGCGCTAC
**ChIP_Hoxc13**	ChIP	Promoter	AGACCCAGGCTTAGCATCAC; TAAGAAATCCGGCGACTCC
**ChIP_Hoxa10**	ChIP	Promoter	TAGCCCTTTCTGGCTGACAT; ACGAAACCAAACTGGGAGTG
**ChIP_Hoxa11**	ChIP	Promoter	GAAGGGAGGCTGGAGAAATC; AGCCCAATGATGGATTTTGA
**ChIP_Vax2**	ChIP	Promoter	CCTCCCCTTGTCTCTCTTCC; CTGGGGTTCCAGGACTCTG
**ChIP_Hand2**	ChIP	Promoter	CTCGGCAATTAGCAACGTG; CGCTCGGGTTAATATATGTCG
**ChIP_Wnt10a**	ChIP	Promoter	TTCAACCAGGAGGGTGAGAG; GGGTGCTTTGAGACATGACC
**ChIP_Chdh**	ChIP	Promoter	TCAGCTAAACGGATGGGAAC; GGGCCATGTTGCTAGTGTTG
**ChIP_Pcdhb18**	ChIP	Intragenic CGI	CAGACCGAGGCTCACCTG; ATCGGGTAGAGCACGAAGG
**ChIP_Pcdhb20**	ChIP	Intragenic CGI	GAACCTGGCTACCTGGTCAC; CCTGGCTCTGTAGCCTTGAG
**ChIP_intergenic_1**	ChIP	chr17:10557867-10558579	CCGGTGAATTCGGATTCTTA; TGTGCTAGCTTTGTGCGTAA
**ChIP_intergenic_2**	ChIP	chr3:79894156-	AAAAACCGGGTCATCTCTCTT; TTCCCCGAAAACTAAAGACG

		79894866	
**BS_Hoxc9**	BS	Promoter	GTTTGTTTGTTTGTTTGAGATTTT; CCCAAAACCAAAATTACCCTATACT
**BS_Hoxc9 (nested)**	BS	Promoter	GATAATTTAGGTTGGGAGGGAGTAT; CCCTATACTCTAATTCAATAACTCTAAAC
**BS_Hoxc10**	BS	Promoter	GGAGTTATAGGTTGATTTTTTAGT; AAAAACTAACATCTTTTTTCCCC
**BS_Hoxc10 (nested)**	BS	Promoter	TATAGGTTGATTTTTTAGTAAATAAG; ACTAACATCTTTTTTCCCCCATC
**BS_Hoxc13**	BS	Promoter	GTTTTTAAAAAGTTGGAGTAG; TAACTACCCCCAAATAAATAAC
**BS_Hoxc13 (nested)**	BS	Promoter	AAGTTGGAGTAGATTATGTTATGA; CAAATAAATAACCATAACCC
**BS_Hoxa10**	BS	Promoter	GTGTAAATTAGAGTGGGTGGT; CAAAACATCAAAAAAAAAAACT
**BS_Hoxa10 (nested)**	BS	Promoter	GTGGAGTTTAGGATTYGTTTTTTT; CAAAAAAAAAAACTCCTACTACC
**BS_Vax2**	BS	Promoter	TAGGGAGGGATTTGTTATTTT; CAAAAAAAACAACTTAATAACCC
**BS_Vax2 (nested)**	BS	Promoter	TTATTTTGAGTTAGGATTAG; ACAACTTAATAACCCAAAAAA
**BS_Hand2**	BS	Promoter	ATTGGGGGGAAATATTTTAA; TCCRAACAAAAATCTTAAACCCT
**BS_Hand2 (nested)**	BS	Promoter	TTATGGTTGATAAAAGGGTT; TCTTAAACCCTTAAAAACAC
**BS_Wnt10a**	BS	Promoter	AGTATGTTATTTTTTGTGGTGA; ACACAACTCCTACTCAAATC
**BS_Wnt10a (nested)**	BS	Promoter	GAATTTTTAGATATTATATGGGGG; ACTCCTACTCAAATCACAACT
**BS_Pcdhb18_CGI**	BS	Intragenic CpG island	TTATATTATTTATTYGTTGTTGTTA; AACAACCAAACATTCTATCCA
**BS_Pcdhb18_CGI (nested)**	BS	Intragenic CpG island	GTTAATTTAAGATTTATAGATG; AACCAAACATTCTATCCAAA
**BS_Pcdhb20_CGI**	BS	Intragenic CpG island	TAAAGATTTAGGTTTTAATG; ACCAACCTATACTTAAACACAT
**BS_Pcdhb20_CGI (nested)**	BS	Intragenic CpG island	GGTTTTAATGTTTATATTATTTATT; CTAACTCTATAACCTTAAAC
**BS_intergenic_1**	BS	chr17:10557961 -10558364	GTTTATGTGTTAGATAAGTAGGTA; AATCCCACCAAAAAAAAACT
**BS_intergenic_1 (nested)**	BS	chr17:10557961 -10558364	GTTGGATTATTTTATTTTTATTTAG; AAAAAACTATCTCTCAACAACCT
**BS_intergenic_2**	BS	chr3:79894354 -79894729	GTGATAAAGAATTAGTATTGAG; CATAACAAACTTAAAAAAAC
**BS_intergenic_2 (nested)**	BS	chr3:79894354 -79894729	AAAGAATTAGTATTGAGATTAT; AACACCACCAAATTAATTTC

Table S1 - Primer sequences used in this study. RT=qRT-PCR, BS=bisulfite sequencing. For bisulfite sequencing some primers contain mixed bases. Y=Pyrimidine (C or T), R=Purine (G or A).

Table S2. Number of reads mapped in ChIP-seq and mRNA-seq experiments

Experiment	Sample	Type of sequencing	Number of mapped reads
**ChIP-seq**	H3K27me3 *Dnmt1+/+*	36bp single-end<br>Illumina Genome Analyser II	36,488,593
	H3K27me3 *Dnmt1-/-*		36,062,432
	H3K4me3 *Dnmt1+/+*		36,751,459
	H3K4me3 *Dnmt1-/-*		35,931,255
**mRNA-seq**	*Dnmt1+/+*	50bp single end<br>Illumina HiSeq2000	56,211,625
	Dnmt1-/-		60,072,435
